# From structural design to delivery: mRNA therapeutics for cancer immunotherapy

**DOI:** 10.1002/EXP.20210146

**Published:** 2023-11-17

**Authors:** Feng Zhou, Lujia Huang, Shiqin Li, Wenfang Yang, Fangmin Chen, Zhixiong Cai, Xiaolong Liu, Wujun Xu, Vesa‐Pekka Lehto, Ulrich Lächelt, Rongqin Huang, Yang Shi, Twan Lammers, Wei Tao, Zhi Ping Xu, Ernst Wagner, Zhiai Xu, Haijun Yu

**Affiliations:** ^1^ State Key Laboratory of Chemical Biology and Center of Pharmaceutics, Shanghai Institute of Materia Medica Chinese Academy of Sciences Shanghai China; ^2^ University of Chinese Academy of Sciences Beijing China; ^3^ The United Innovation of Mengchao Hepatobiliary Technology Key Laboratory of Fujian Province Mengchao Hepatobiliary Hospital of Fujian Medical University Fuzhou China; ^4^ Department of Applied Physics University of Eastern Finland Kuopio Finland; ^5^ Department of Pharmaceutical Sciences University of Vienna Vienna Austria; ^6^ Department of Pharmaceutics, School of Pharmacy, Key Laboratory of Smart Drug Delivery Ministry of Education, Fudan University Shanghai China; ^7^ Department of Nanomedicine and Theranostics, Institute for Experimental Molecular Imaging RWTH Aachen University Clinic Aachen Germany; ^8^ Center for Nanomedicine and Department of Anaesthesiology, Brigham and Women's Hospital Harvard Medical School Boston Massachusetts USA; ^9^ Institute of Biomedical Health Technology and Engineering and Institute of Systems and Physical Biology Shenzhen Bay Laboratory Shenzhen China; ^10^ Pharmaceutical Biotechnology, Center for Nanoscience Ludwig‐Maximilians‐Universität Munich Germany; ^11^ School of Chemistry and Molecular Engineering East China Normal University Shanghai China

**Keywords:** cancer immunotherapy, cell‐targeted delivery, mRNA design, mRNA therapeutics, organ‐specific delivery

## Abstract

mRNA therapeutics have emerged as powerful tools for cancer immunotherapy in accordance with their superiority in expressing all sequence‐known proteins in vivo. In particular, with a small dosage of delivered mRNA, antigen‐presenting cells (APCs) can synthesize mutant neo‐antigens and multi‐antigens and present epitopes to T lymphocytes to elicit antitumor effects. In addition, expressing receptors like chimeric antigen receptor (CAR), T‐cell receptor (TCR), CD134, and immune‐modulating factors including cytokines, interferons, and antibodies in specific cells can enhance immunological response against tumors. With the maturation of in vitro transcription (IVT) technology, large‐scale and pure mRNA encoding specific proteins can be synthesized quickly. However, the clinical translation of mRNA‐based anticancer strategies is restricted by delivering mRNA into target organs or cells and the inadequate endosomal escape efficiency of mRNA. Recently, there have been some advances in mRNA‐based cancer immunotherapy, which can be roughly classified as modifications of the mRNA structure and the development of delivery systems, especially the lipid nanoparticle platforms. In this review, the latest strategies for overcoming the limitations of mRNA‐based cancer immunotherapies and the recent advances in delivering mRNA into specific organs and cells are summarized. Challenges and opportunities for clinical applications of mRNA‐based cancer immunotherapy are also discussed.

## Graphical abstract

Systemic or topical mRNA delivery for immune function modulation is emerging as a promising option for cancer immunotherapy. In this review, strategies for enhancing mRNA‐based cancer immunotherapy from the perspective of mRNA structure design and delivery systems are first summarized. Advances of delivering mRNA into specific organs or cells for cancer treatment and opportunities in clinical translation are discussed.

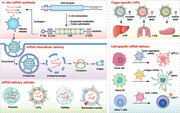

## 1 INTRODUCTION

Tumor cells escape immune surveillance, snatch nutrients, and inhibit surrounding cells from fulfilling normal functions, which seriously impair human health. Conventional cancer therapeutic modalities such as surgical resection, radiotherapy, and chemotherapy are frequently inadequate to eradicate tumor cells, coupled with their unwanted damage to normal cells. As such, utilizing the killing effect of immune system on tumor cells by enhancing innate or adaptive immune response, called cancer immunotherapy, has recently become a promising anti‐tumor strategy.^[^ ^1^ ^]^ Compared to the conventional therapy approaches, immunotherapy elicits tumor‐specific immune response to regress tumor growth and even cure certain cancer types.^[^ ^2^ ^]^ However, there are also some bottlenecks limiting the development of cancer immunotherapy. For example, cancer immunotherapy is not practical for all patients, partly due to the heterogeneity of cancer types and patient populations like the varieties of cumulative oncogene mutations, the states of immune cells and tumor size.^[^ ^3^ ^]^ Moreover, cancer immunotherapy generally requires longer treatment cycles and possesses limited efficacy, which usually needs a combination with other strategies, such as mRNA therapeutics.^[^ ^4^ ^]^

mRNA is a kind of single‐stranded ribonucleic acid transcribed from a DNA template. It is a bridge connecting genes and proteins, carrying genetic information and guiding protein synthesis in the cytoplasm. mRNA‐based therapeutics were previously exploited to regulate protein expression by locally injecting mRNA, but this approach exhibited restricted protein expression efficiency and limited potential for clinical application.^[^ ^5^ ^]^ In 1978, Dimitriadis et al. delivered mRNA encoding rabbit globulin to mouse lymphocytes using liposomes and produced functional proteins, opening the prelude for mRNA delivery in vivo.^[^ ^6^ ^]^ Many recent reports revealed mRNA could be applied in various fields like protein replacement therapy, vaccines, gene editing, and cellular reprogramming^[^ ^7^ ^]^ for treating a diverse spectrum of diseases like infectious diseases,^[^ ^8^ ^]^ rare genetic disease,^[^ ^9^ ^]^ and cancer.^[^ ^10^ ^]^ Moreover, the US Food and Drug Administration (FDA) approval for the clinical application of two mRNA‐based vaccines from Pfizer‐BioNTech and Moderna in 2020 to prevent COVID‐19 infection ushered a boom in the development of mRNA‐based treatments of many diseases, including cancer.^[^ ^11^ ^]^

Profiting from the scale‐up manufacturing technique, alongside superiorities of presenting complete epitopes and multi‐antigens to APCs, mRNA products have high propensity of replacing protein‐based immunotherapies in the future.^[^ ^11^, ^12^ ^]^ Such mRNA‐based platforms can simultaneously encode several full‐length antigens to stimulate a broader adaptive immune response, thus possessing the potential of eradicating tumors. As regards the action mechanism, the mRNA immunotherapeutic strategy utilizes internal organelles and molecules (e.g., ribosomes, enzymes, amino acids) to biosynthesize target proteins under the guidance of delivered mRNA provided it is released in the host cell cytoplasm.^[^ ^13^ ^]^ On the aspect of efficacy and clinical transformation, a low dosage of mRNA could generate sufficient antigens to induce a potent immune response against tumors. Moreover, with the maturation of mRNA manufacturing techniques and in vitro transcription (IVT), large‐scale and pure mRNAs can be produced with low batch‐to‐batch variation.^[^ ^14^ ^]^ mRNA‐based cancer immunotherapy also displays satisfying biosafety due to the relatively moderate killing process of tumor cells without affecting non‐malignant cells. Compared with DNA, mRNA functions without entering the nucleus, and after cytoplasmatic translation into proteins is accomplished, it can be degraded by ribonucleases (RNases), preventing the risk of genome integration and permanent cellular reprogramming.^[^ ^15^ ^]^

Despite the promising potential of mRNA‐based cancer immunotherapy, its clinical translation is impeded by several bottlenecks. For example, mRNA with a single‐stranded structure is liable to degradation in biological media. The innate immunogenicity of mRNA is paradoxically beneficial and detrimental to the patient, which is partly determined by the delivery platforms and the purity of mRNA (e.g., dsRNA produced during IVT process)^[^ ^16^ ^]^ as well as the RNA design (e.g., RNA modifications). Besides, the inadequate transfection efficiency of mRNA and the barrier of delivering mRNA to target cells also restrict its development.^[^ ^17^ ^]^ These shortcomings hinder the target cells from producing adequate amounts of protein for effective immune response against tumors. Strategies are therefore needed for optimum release of mRNA in the target cells to produce sufficient aimed proteins for improved cancer immunotherapy.^[^ ^18^ ^]^

In recent years, studies of mRNA in vivo application and clinical trials have been booming, owing to the processing of mRNA‐producing methods and advanced drug‐delivery platforms.^[^ ^19^ ^]^ IVT method ensures production of mRNA similar to naturally matured transcripts, while decreasing the innate immunogenicity of extraneous mRNA. Profiting from the development of precision medicine, targeted therapy, and exploitation of abundant excellent biomaterials, organ‐ and/or cell‐targeted delivery of mRNA can be achieved practicably, ensuring that target cells produce desired proteins.^[^ ^20^ ^]^ These new technologies minimize the limitations of mRNA immunotherapy and expand its application to different cancers.^[^ ^8^ ^]^ However, despite the rapid advances in mRNA immunotherapy for cancer, there is still a considerable gap between laboratory studies and clinical translation, which limits benefits to cancer patients at the current stage.^[^ ^21^ ^]^ As such, a follow‐up of current developments in mRNA immunotherapy, especially the latest excellent delivery systems targeting specific organs or cells and new attempts of co‐delivering mRNA with other cargos like adjuvants is crucial.^[^ ^22^ ^]^

**FIGURE 1:**
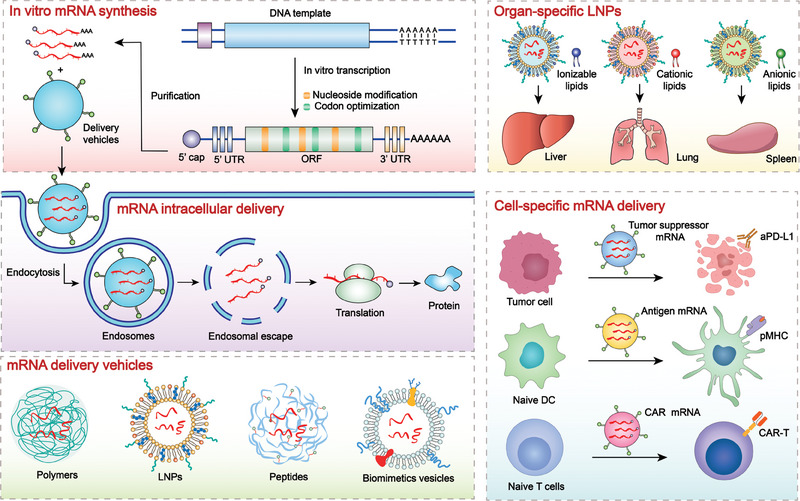
Schematic illustration of mRNA therapeutics for precise cancer immunotherapy. First, in vitro transcribed mRNA are optimized and modified for improved stability, translation efficiency and lower immunogenicity before being encapsulated into different delivery vehicles. Second, mRNA‐encoding diverse proteins like tumor‐suppressing factors, antigens, chimeric antigen receptor (CAR), T‐cell receptor (TCR), and cytokines are supposed to be transported to specific organs and cell types and escape from endosomes into cytoplasm to express proteins for subsequent precise anti‐tumor immunotherapy.

This review therefore focuses on strategies to overcome the bottlenecks of mRNA‐based cancer immunotherapy, such as mRNA instability, innate immunogenicity, and low transfection efficiency. Moreover, advances in designing optimal delivery platforms to transport mRNA to specific sites are emphasized, as classified by the target organs and cells (Figure 1). The functions of particular molecular structures in the delivery system, especially mRNA carriers with target‐delivery properties are also discussed. These discussions are significant as mRNA immunotherapy has emerged as a time‐transgressive strategy in cancer treatment.

## 2 MOLECULAR DESIGN FOR MRNA‐BASED CANCER IMMUNOTHERAPY

mRNA is a negatively charged, single‐stranded RNA, which contains genetic information transcribed from DNA and relies on ribosomes to translate into proteins for specific life functions.^[^ ^13a^ ^]^ This fragile macromolecular structure determines the instability of mRNA in internal body environment rich in various peptides and enzymes. Besides, polyanionic mRNA repels the negatively charged cell membrane (CM), making it hard to enter the cell. IVT method harnesses bacteriophage T7 RNA polymerase (T7 RNAP) to synthesize mRNA with high fidelity under the guidance of the linearized DNA template. The obtained mRNA consists of three components: one open‐reading fragment (ORF), the five‐prime (5′), and the three‐prime (3′) untranslated regions (UTRs). Although the IVT method greatly increases the purity and quality of mRNA, the protein expression of mRNA is not effective enough due to the gap between naturally derived and synthetic mRNAs, which requires further modification of mRNA structure and nucleotides. Moreover, the low efficiency of endosomal escape significantly impairs the efficacy of RNA drugs. For example, the FDA‐approved DLin‐MC3‐DMA LNP can only mediate 1–4% RNA release into the cytoplasm.^[^ ^23^ ^]^

The innate immunogenicity of mRNA is another issue, which should be carefully considered. Extraneous mRNA acts as an immunogen rather than a therapeutic drug, which is determined by the innate immunogenicity of mRNA.^[^ ^24^ ^]^ APCs particularly recognize IVT mRNA via pattern recognition receptors (PRRs) such as toll‐like receptors (TLRs) to stimulate secretion of type I interferon (IFN‐I, including IFN‐α and IFN‐β) and proinflammatory factors that significantly impair protein expression process of mRNA. Moreover, IFN‐I behaves paradoxically as beneficial and detrimental to cancer immunotherapy.^[^ ^25^ ^]^ It promotes dendritic cells (DCs) maturation, antigen presentation to T cells, and activation of CD8^+^ T cells.^[^ ^26^ ^]^ On the other hand, IFN‐I can increase the number of Treg and Th17 cells and induce the intratumoral infiltration of myeloid‐derived suppressor cells (MDSC) to promote the immune escape of tumor cells.^[^ ^27^ ^]^

Notably, the production of double‐stranded RNA (dsRNA) during the mRNA IVT process increases the risk of generating harmful innate immune responses. The recognition of dsRNA by oligoadenylate synthetase (OAS), TLR3, retinoic acid‐inducible gene I (RIG‐I), melanoma differentiation‐associated protein 5 (MDA5), and RNA‐dependent protein kinase (PKR) causes RNA degradation and hinders the production of antigens, thus impeding immune response for tumor cell killing.^[^ ^28^ ^]^ A strict RNase III digestion method has been employed to remove dsRNA in the IVT mRNA product.^[^ ^24^, ^29^ ^]^ Dousis et al. designed a double mutant of T7 RNAP that could produce highly pure IVT mRNA and less immunostimulatory byproducts such as dsRNA, which could accelerate the industrial production of mRNA.^[^ ^30^ ^]^ In the following sections, strategies to overcome the above bottlenecks of mRNA‐based cancer immunotherapy such as structure modification, nucleoside modification, codon optimization of mRNA, adjuvants application, and delivery system optimization are discussed.

### 2.1 Structural modification of mRNA

In the initial stage of in vivo mRNA therapy, mRNA injected into the body will cause a series of heterologous immune responses and be cleaved by the immune system, as though the body is fighting against virus invasion, which dramatically limits the application of mRNA therapy. Therefore, technological breakthroughs are essential to overcome mRNA immunogenicity. In 2005, Katalin et al. found that replacing the uridine with pseudouridine could reduce the risk of DC activation by mRNA, not only protecting synthetic mRNA from immune elimination but also significantly enhancing the efficiency of protein expression.^[^ ^31^ ^]^ This discovery addressed the concerns of mRNA‐based therapy and thus opened the prelude of mRNA clinical application. In addition to nucleoside replacement, IVT‐mRNA structural modification includes the five‐prime cap (5′Cap), poly (A) tail, and UTRs remolding. Although intracellular naturally‐produced mRNA consists of 5′Cap and poly (A) tail, IVT method requires additional steps for capping and polyadenylation of mRNA.

The 5′Cap of mRNA consists of 7‐methylguanosine (m7G) and is attached to mRNA through a 5′−5′‐triphosphate bridge. It regulates the initiation of mRNA translation by binding to translation initiation factor 4E (eIF4E). Therefore, the stable presence of 5′Cap on mRNA and the efficient interaction with eIF4E is beneficial to the translation process of mRNA. Tan et al. linearized luciferase‐encoding plasmids with different structures and transcribed them to obtain mRNAs with different cap structures.^[^ ^32^ ^]^ They proved that adding a cap structure can significantly improve the protein expression efficiency of mRNA. Wojtczak. et al. synthesized a series of dinucleotide cap (m7GpppG) analogs containing a 5′‐phosphorothioate (5′‐PSL) moiety.^[^ ^33^ ^]^ The synthetic 5′Cap possesses low sensitivity to decapping enzymes and sufficient affinity for eIF4E, ensuring excellent protein expression efficiency of mRNA. Shanmugasundaram et al. summarized the recent chemically modified mRNA cap analogs applied in mRNA vaccines to improve mRNA's translational process, which can facilitate the clinical transformation of mRNA.^[^ ^34^ ^]^

Apart from synthetic 5′Cap, modification of existing 5′Cap can also improve the protein expression efficiency of mRNA. Dülmen et al. demonstrated that site‐specific chemical enzymatic conversion of the 5′Cap can regulate the translation process of mRNA and generate an approximately threefold higher antiviral immune response in human cells.^[^ ^35^ ^]^ They observed the same results when using the mRNA of receptor binding domain (RBD) of SARS‐CoV‐2, proving that such an enzymatic modification of 5′Cap is expected to advance the mRNA‐based therapy of COVID‐19 and cancer.^[^ ^35^ ^]^ Modifying the base of 5′Cap can also improve the stability of mRNA. Mauer et al. discovered a nucleotide N6,2′‐O‐dimethyladenosine (m6Am) for base modification of 5′Cap, which can enhance the resistance of mRNA against decapping enzyme DCP2, thereby increasing mRNA stability.^[^ ^36^ ^]^ Besides, when designing the mRNA sequence, highly stable secondary structures and hairpin loops should be avoided, which reduce the transfection efficiency of mRNA.^[^ ^37^ ^]^

Considering the importance of 5′Cap on IVT mRNA, assessing the presence and position of the 5′cap on mRNA represents a general quality control for mRNA‐based therapeutics. Vlatkovic et al. developed ribozyme cleavage‐based assays to estimate the capping efficiency of mRNA with different features.^[^ ^38^ ^]^ They found that mRNAs with diverse structures possessed altered capping efficiency, which should be considered when adding 5′Cap onto mRNA.

**FIGURE 2:**
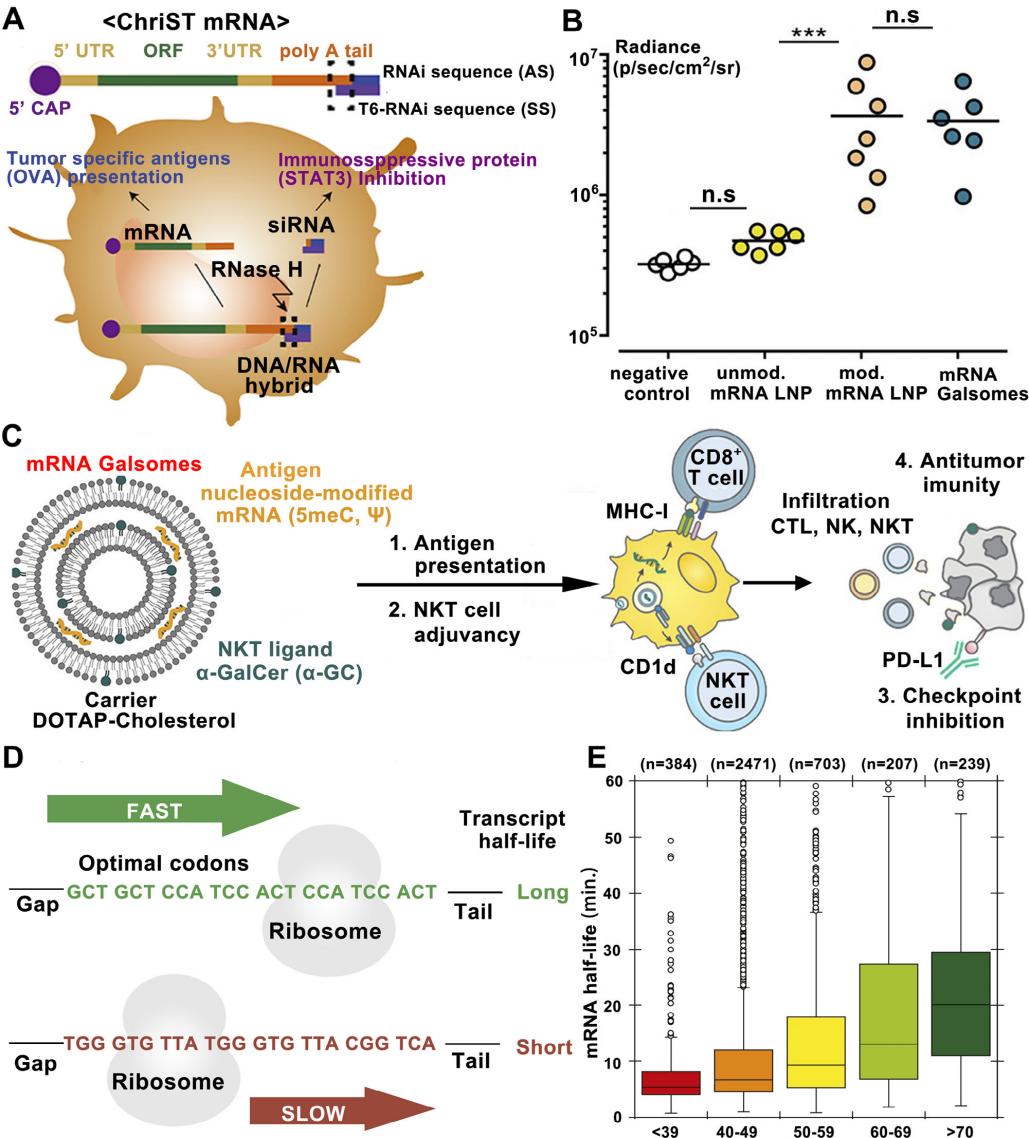
Schematic illustration of mRNA structural modification, adjuvant adding, nucleoside modification, and codon optimization for improved mRNA‐based cancer immunotherapy. A Schematic illustration of the preparation and application of ChriST mRNA in DCs‐targeted cancer immunotherapy. Reproduced with permission.^[^ ^39^ ^]^ Copyright 2020, Elsevier. B The expression levels of fLuc mRNA in vivo with modified and unmodified mRNA and other treatments. C Adjuvant α‐GalCer and nucleoside modification in promoting the stability of mRNA and improving the antigen‐presenting ability of DCs. Reproduced with permission.^[^ ^45^ ^]^ Copyright 2019, American Chemical Society. D Schematic illustration of codon optimization in prolonging the transcript half‐life of mRNA. E Box plot of mRNA stability with optimal codons percent. Reproduced with permission.^[^ ^46^ ^]^ Copyright 2015, Elsevier.

Adding a 3′ poly(A) tail on mRNA can also significantly decrease the rate of exonuclease degradation and improve mRNA stability. The mRNA synthesized by Tan et al. has an extended 3′poly(A) tail structure, which improves the stability and protein expression efficiency of mRNA.^[^ ^32^ ^]^ Lee et al. synthesized mRNA with 5′Cap and poly (A) tail structure and added small interfering RNA (siRNA) that can induce STAT3 gene silencing to the 3′poly (A) tail of mRNA through base complementation.^[^ ^39^ ^]^ In the intracellular environment, the mRNA‐siRNA complex can be cleaved by RNase H to yield mRNA and siRNA. Specifically, the released mRNA translates into tumor‐specific antigens to induce DC maturation and the siRNA inhibits STAT3 gene (a kind of immunosuppressive factor that interferes with the successful DC maturation) for cancer therapy^[^ ^40^ ^]^ (Figure 2A). The study suggests manipulating the structure of nucleotide chain to synthesize multifunctional mRNA is expected to break the deadlock in cancer treatment. Moreover, poly (A) tail length is significant for mRNA translation efficiency, and the most suitable length of poly (A) tail varies in different cells. A short poly (A) tail will not effectively protect mRNA from exonuclease degradation. A long poly (A) tail may cause poly (A) binding protein to bind to 5′Cap through translation initiation factors such as eIF4E and eIF4G.^[^ ^37b^ ^]^ As a result, the mRNA forms an end‐to‐end closed‐loop structure, affecting its protein expression efficiency.^[^ ^41^ ^]^ Therefore, choosing a poly (A) tail of appropriate length is crucial to improve mRNA stability and maintain protein expression efficiency.

Another strategy to improve the stability of mRNA and protein expression efficiency is optimizing UTRs sequence. UTRs are located on both sides of the coding sequence, do not encode proteins and interact with RNA‐binding proteins to process ORF translation. UTRs of mRNA can be divided into 5′ and 3′ sequences. The 5′ UTRs are close to the start codon of mRNA and can influence the rate of ORF translation. A report showed that shorter 5′ UTRs without complex secondary structures and start codons like AUG and CUG are beneficial for initiating the translation process of mRNA.^[^ ^42^ ^]^ These points are worthy of consideration when designing mRNA vaccines. The 3′ UTRs are commonly regulatory elements, which also impact the expression efficiency of mRNA. Alexandra et al. screened and obtained several novel 3′ UTRs, significantly improving the protein translation level of mRNA compared to the general 3′ UTRs.^[^ ^42^ ^]^ Zeng et al. identified the optimal combination of 5′ and 3′ UTRs by analyzing the endogenous gene expression and designing UTRs sequences innovatively, which were five‐ to tenfold more efficient for protein expression than endogenous UTRs.^[^ ^43^ ^]^ Besides, machine learning can be applied to facilitate UTRs sequence designing. Castillo‐Hair et al. developed a convolutional neural network model trained on the experimental data named Optimus 5‐Prime, which can be combined with algorithms to design de novo UTRs sequences with improved translation efficiency, accelerating the process of exploiting novel UTRs in mRNA therapeutics.^[^ ^44^ ^]^

The rational manipulation of the above elements (5′Cap, poly (A) tail, UTRs) in the mRNA structure not only significantly improves the stability and protein expression efficiency of mRNA, but also regulates the adjuvant effect of mRNA to balance its innate and adaptive immunity.^[^ ^37b^ ^]^ Therefore, it is necessary to rationally design a proper mRNA structure for improved mRNA‐based cancer therapy. However, in different species and cell types, the performance of element optimization of mRNA varies a lot.^[^ ^37b^ ^]^ Considering the pharmacology in target cells, specific mRNA structures should be designed for different species and cell types.

### 2.2 Nucleoside modification of mRNA

Foreign mRNAs with natural nucleotides (such as uridine and cytidine) are intended to be identified and combined by intracellular proteins (e.g., PKR, RIG‐I) which protect cells from the invasion of outer mRNAs by inhibiting the translation process. For this reason, researchers incorporated artificially modified nucleotides into mRNA for improving the resistance of mRNA to intracellular molecules.^[^ ^47^ ^]^ 5‐methylcytidine (m5C), N6‐methyladenosine (m6A), pseudouridine (Ψ), 5‐methoxyuridine (5moU), and 1‐methylpseudouridine (m1 Ψ) are the primary modified‐nucleotides that have been used for mRNA remolding, which not only improves protein translating efficiency but also reduces the innate immune activation of mRNA.^[^ ^48^ ^]^ Jeught et al. demonstrated that N1 methylpseudouridine‐modified mRNA delivered by lipoplexes induced potent antitumor T cell immunity with improved inflammatory safety.^[^ ^49^ ^]^ Liu et al. synthesized mRNA encoding cytokines with complete substitution of UTP by pseudouridine‐5′‐triphosphate, substantially improving the stability of mRNA and translational efficiency.^[^ ^50^ ^]^

Similarly, Huang et al. replaced the UTP of mRNA with N1‐Me‐Pseudo UTP and obtained mRNA with higher stability and lower innate immunogenicity.^[^ ^51^ ^]^ They delivered mRNA encoding the bispecific antibody of CD3 and B7 homolog three protein (a checkpoint molecule) with LNP and achieved high serum antibody levels to induce durable antitumor efficacy against hematologic malignancies and melanoma. Moreover, nucleoside modification can also minimize the immune recognition of extraneous mRNA, thus reducing the release of IFN‐I which prematurely hinders mRNA translation. Verbeke et al. modified mRNA nucleosides with 5meC and Ψ and compensated the loss of RNA's self‐adjuvant‐effect with adjuvant α‐galactosylceramide (α‐GC)^[^ ^45^ ^]^ (Figure 2B,C) or monophosphoryl lipid A (MPLA)^[^ ^52^ ^]^ respectively, where the mRNA‐based therapeutic platforms displayed reduced IFN‐I production and substantially enhanced protein expression levels in vivo. However, a report showed nucleoside modification of mRNA may impair the efficacy of cancer immunotherapy and should be considered when designing mRNA‐based therapeutics.^[^ ^53^ ^]^

### 2.3 Codon optimization of mRNA

Codon optimization generally refers to adjusting the protein‐coding regions (like ORF) in mRNA. The smooth interpretation of the codon information in ORF is closely related to the protein expression efficiency of mRNA.^[^ ^54^ ^]^ Clearing the barriers of codon interpretation and adding elements that facilitate interpretation will enhance the protein expression efficiency of mRNA. There are several reasons that optimal codons can facilitate the translation process of mRNA. First, codons with rich tRNA abundance in the cytoplasmic pool can recruit amino acids quickly, thus accelerating the translation rate.^[^ ^46^, ^55^ ^]^ Second, optimal codons with flexible construction facilitate the process of ribosome translocation and regulate the translation elongation rate.^[^ ^55^, ^56^ ^]^ Moreover, reports showed that the uridine‐rich regions in ORF can bind and activate RIG‐I (a member of the RNA helicase family of DexD/H boxes), leading to the premature halt of mRNA translation, which should be avoided when selecting codons.^[^ ^57^ ^]^ These reports revealed that optimal codons in mRNA improve the protein expression efficiency through facilitating ribosome translocation, amino acids transporting by tRNA and avoiding the premature stop of translation.

mRNA with optimal codons also possesses higher stability. Presnyak et al. demonstrated that optimal codons significantly prolonged the half‐life of mRNA by substituting non‐optimal codons (Figure 2D,E). They also constructed a metric for describing codon occurrence to mRNA stability correlation coefficient and found a series of optional codons with appropriate proportions for stable mRNA preparation.^[^ ^46^ ^]^

Optional codons can be discovered from naturally stable mRNAs or mRNAs encoding naturally highly expressed proteins in the target cells. This emphasizes the species and cell heterogeneity of preferred codon types and proportions for mRNA design. Yang et al. substituted non‐optimal codons with synonymous codons of mRNA encoding erythropoietin (EPO) based on the principles of codon usage preference and frequency in different cell types, the requirement of avoiding specific restriction enzyme cutting sites, GC content, etc.^[^ ^58^ ^]^ The results showed that the EPO expression efficiency with codon‐optimized mRNA was significantly higher than unoptimized mRNA in human hepatocellular carcinoma (HCC) cells. Therefore, designing mRNA with the best types and proportions of codons and with less stiffened secondary structures, uridine‐rich sites, etc., can improve the protein expression efficiency of mRNA, which might promote the clinical translation of mRNA therapeutics for cancer immunotherapy.

However, due to the degeneracy of codons, there are hundreds of millions of codon combinations and secondary structures of the same protein amino acid sequence, leading to the time‐consuming and laborious screening of mRNA nucleotide sequences with the best stability and translation efficiency. Recently, an artificial intelligence (AI) and dynamic programming algorithm named LinearDesign has been developed to shorten this screening process, allowing for the discovery of mRNA with the best sequence in about ten minutes.^[^ ^59^ ^]^ Results showed that LinearDesign substantially improved mRNA half‐life and protein expression by exploring previously unreachable but highly stable and efficient mRNA sequence areas.^[^ ^59^ ^]^ The report reveals the great potential of AI‐facilitated sequence designing in mRNA medicine encoding all therapeutic proteins.

### 2.4 Adjuvant application in mRNA‐based cancer immunotherapy

Adjuvants are organic or inorganic molecules used alone or combined with other immunotherapeutic platforms, particularly in the preparation of cancer vaccines to enhance immune response. According to the pathways stimulated, adjuvants can be roughly classified into three main types, agonists of TLRs, NOD‐like receptors (NLRs), and stimulators of IFN genes (STING, an intracellular receptor residing in the endoplasmic reticulum).^[^ ^60^ ^]^ Essentially, adjuvants are agonists of different signaling pathways involved in the immune response. After entering the circulatory system, adjuvants can activate APCs and facilitate the process of antigen presentation on MHC molecules, which is crucial to enhance cellular immunity against tumor cells.^[^ ^61^ ^]^

The agonists of TLRs are the most widely used adjuvants in mRNA‐based cancer immunotherapy. For example, Lee et al. incorporated tri‐palmitoyl‐S‐glyceryl cysteine‐modified pentapeptide (Pam3, the agonist of TLR‐1/2) into an mRNA vaccine via hydrophobic interaction between the lipid tails of Pam3 and the lipid components of LNPs.^[^ ^62^ ^]^ This system can be recognized by different subclasses of TLRs during the cellular uptake of LNPs and synergistically enhance the antitumor immune response. Poly‐IC, the agonist of TLR3, has also been used as an adjuvant in mRNA therapeutics to boost the synergic effect of DC vaccination and radiotherapy, which obtained curative effects in advanced cancer patients.^[^ ^63^ ^]^ Verbeke et al. co‐delivered TLR4 agonist monophosphoryl lipid A (MPLA) and mRNA with LNP to induce strong T‐cell immunity against tumor cells.^[^ ^52^ ^]^ Besides, MPLA compensated for the reduced efficiency of DC activation due to mRNA nucleoside modification (5meC, Ψ). Gardiquimod, a hydrophobic TLR7 agonist, was loaded into a poly (lactic‐*co*‐glycolic acid) (PLGA)‐based mRNA delivery NP.^[^ ^64^ ^]^ The obtained mRNA platform effectively activated DCs and cytotoxic T cells and markedly inhibited tumor growth. Moreover, the hydrophobic agonist of TLR7/8 (Resiquimod, R848) has been widely used as a pulsation adjuvant in mRNA vaccine after being modified with palmitic acid or encapsulated by graphene oxide (GO) or polymer nanoparticle^[^ ^65^ ^]^ to improve its physical property.^[^ ^66^ ^]^

α‐GC is a well‐known glycolipid antigen that possesses an indirect adjuvant effect. It can be presented on the MHC‐I‐like molecule of APCs (CD1d) and interacts with natural killer T cells, thus eliciting the production of cytokines and activation of NK cells. For example, α‐GC was used as an immune adjuvant in different studies to pulse mRNA therapy and induced strong antitumor therapeutic effects.^[^ ^45^, ^67^ ^]^

STING agonists have been widely employed as immune adjuvants to enhance antitumor immunity through inducing cytokines and chemokines, including IFN‐I.^[^ ^68^ ^]^ Recently, numerous natural and synthetic STING agonists, such as cyclic GMP‐AMP^[^ ^69^ ^]^ and cyclic dinucleotides,^[^ ^70^ ^]^ have been reported for cancer immunotherapy.^[^ ^71^ ^]^ To apply STING agonist to the mRNA platform, Miao et al. condensed mRNA with synthetic STING‐activatable lipids.^[^ ^72^ ^]^ The obtained formulation activated STING pathways potently, induced maturation of the antigen‐presenting cells (APCs), and enhanced antitumor efficacy in melanoma tumor models.

Apart from adding an extra adjuvant to the system, delivery materials can also act as a self‐adjuvant for mRNA‐based cancer immunotherapy.^[^ ^24^, ^73^ ^]^ Papachristofilou et al. delivered mRNA encoding six NSCLC‐associated antigens through a delivery system based on cationic protein protamine, which acts as a self‐adjuvant and interacts with TLR7, TLR8, and intracellular RNA sensors to induce strong immune response.^[^ ^22^, ^74^ ^]^ Another classical TLR7/8 agonist, R848, was modified with amino lipids to obtain a self‐adjuvant lipid for mRNA‐LNP construction by Yan et al., which mediated strong antitumor immunity in melanoma tumor mouse models.^[^ ^75^ ^]^ Similarly, STING agonist‐derived novel lipids were also developed to construct mRNA‐LNPs for enhanced cancer immunotherapy.^[^ ^76^ ^]^ Inspired by the discovery that polysaccharides found in microbes are potent activators of DC, Son et al. developed a novel nano‐capsule composed of mannan derived from the microbial to transport mRNA and promote a robust DC activation with antitumor efficacy in vivo.^[^ ^77^ ^]^ The microbial components initiated innate and adaptive immune responses via pathogen‐associated molecular patterns (PAMPs)‐PRRs interaction and transported mRNA with potent loading capacity, representing a promising platform in mRNA‐based vaccine. Zhang et al. synthesized a series of lipid‐like compounds with cationic head groups that could efficiently load mRNA via electrostatic interactions. The LNP can also act as a self‐adjuvant and induce IL‐12 excretion by stimulating the TLR4 signal pathway to strengthen the antitumor effect.^[^ ^78^ ^]^

However, direct activation of these signaling pathways may lead to the apoptosis of T cells and B cells, suggesting that organ‐ or cell‐specific delivery of agonists is necessary.^[^ ^79^ ^]^ With the development of novel adjuvants, selecting proper adjuvants for specific platforms is crucial to improve therapeutic efficacy. In the field of mRNA‐based cancer immunotherapy, choosing optimal adjuvant in compliance with the mechanism of therapeutic mRNA could amplify anti‐tumor efficacy, which should be emphasized for accelerating the clinical transformation of mRNA therapies.

## 3 DELIVERY SYSTEMS FOR MRNA‐BASED CANCER IMMUNOTHERAPEUTICS

There are several motivations to formulate mRNA into proper delivery systems. First, naked mRNA, a negatively charged and hydrophilic single polynucleotide chain, is susceptible to ubiquitous RNases in vivo.^[^ ^13^, ^80^ ^]^ These characters suggest that naked mRNA can hardly reach the target tumor sites, traverse the CM and encode target peptides in cytoplasm. Second, naked mRNA requires encapsulation in delivery platforms to enhance its endosomal escape efficiency, which plays a crucial role in the subsequent antigen‐presentation process.^[^ ^80^, ^81^ ^]^ Third, multi‐functional delivery strategies endow mRNA abilities to target specific organs and cells, activating APCs efficiently, and stimulating immune‐related signaling pathways by adjuvant effects, thus significantly improving anti‐tumor efficacy.^[^ ^81^, ^82^ ^]^ In general, a suitable delivery system can be helpful to overcome the bottlenecks in mRNA‐based cancer immunotherapy, such as targeted delivery, improved transfection efficiency, and enhanced intensity of immune response.^[^ ^83^ ^]^

### 3.1 LNPs for mRNA delivery

Our group previously reviewed the advances in harnessing NPs to remold immunosuppressive tumor microenvironment (ITM) for enhanced cancer immunotherapy, indicating the crucial position of NPs in cancer treatment.^[^ ^84^ ^]^ For mRNA delivery, lipid nanoparticles (LNPs) represent the most widely used transporting system,^[^ ^85^ ^]^ especially after FDA approved the clinical application of two LNP formulations of mRNA vaccines for COVID‐19 prevention.^[^ ^11d,e,g^ ^]^ LNPs generally consist of cholesterol (with strong membrane fusion property for promoting intracellular mRNA uptake and LNP stability in vivo), poly‐(ethylene glycol) (PEG, enhancing LNP stability and prolonging circulation time in vivo), and helper lipids (such as phospholipid with membrane‐integrating potential, which contribute to the stability and delivery efficiency of LNPs).^[^ ^86^ ^]^ Despite the promising performance, LNPs with traditional compositions are hard to achieve the desired mRNA delivery efficiency for cancer immunotherapy.^[^ ^87^ ^]^

**FIGURE 3:**
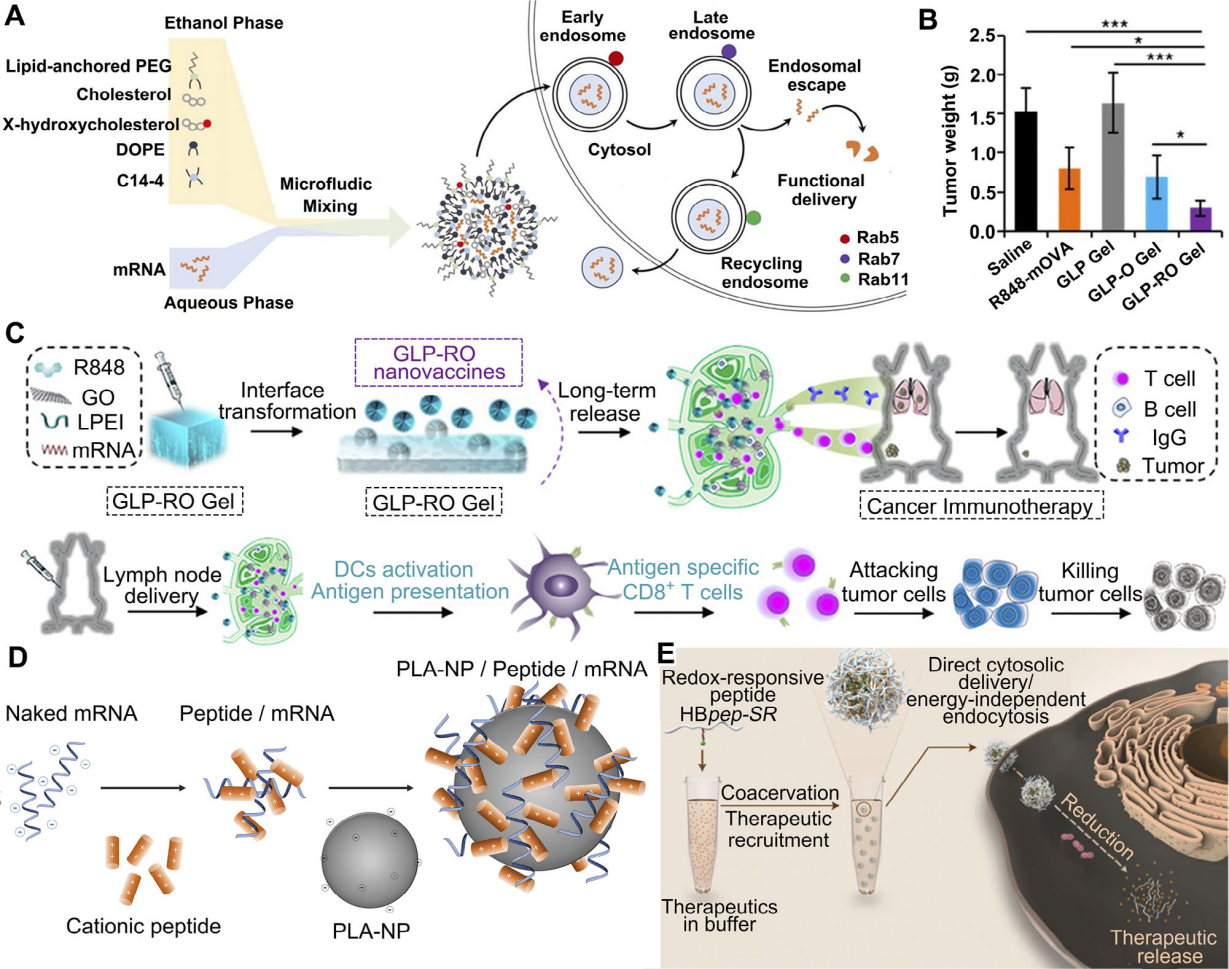
Schematic illustration of LNPs, hydrogel‐based, polymer‐based, and peptide‐based systems for mRNA delivery. A Engineering LNPs with hydroxycholesterol substitution for delivering mRNA to T cells cancer immunotherapy. Reproduced with permission.^[^ ^88^ ^]^ Copyright 2022, Elsevier. B The graph of tumor weight changes with the treatment of GLP‐RO Gel and other groups. C The diagram of GLP‐RO Gel preparation with polyethylenimine and GO hydrogel for durable cancer immunotherapy. Reproduced with permission.^[^ ^66a^ ^]^ Copyright 2021, American Chemical Society. D Schematic diagram of delivering mRNA into DCs with poly lactic acid NPs and CPPs to induce potent immune responses. Reproduced with permission.^[^ ^103^ ^]^ Copyright 2019, Elsevier. E Phase‐separating peptides for mRNA cytosolic delivery with improved stability and high transfection efficiency. Reproduced with permission.^[^ ^104^ ^]^ Copyright 2022, Nature Publishing Group.

To this end, researchers attempted to introduce a series of unique molecules (such as X‐hydroxycholesterol,^[^ ^88^ ^]^ PEG‐lipid,^[^ ^62^, ^78^, ^89^ ^]^ iBL0713 (an ionizable lipid),^[^ ^58^ ^]^ N‐series lipidoids,^[^ ^90^ ^]^ synthetic ionizable lipidoids,^[^ ^72^ ^]^ DOTAP,^[^ ^91^ ^]^ etc.) into LNP compositions to endow them with properties of targeted delivery, high transfection rate, and high endosomal escape rate (Figure 3A). For example, Benedicto et al. found that adding zwitterionic phospholipids containing phosphoethanolamine (PE) head groups into LNPs can significantly enhance the liver‐targeting ability and endosomal escape efficiency of delivered mRNA.^[^ ^92^ ^]^ Besides, adding synthetic amino lipids,^[^ ^81a^ ^]^ unsaturated thiols‐modified ionizable lipids,^[^ ^93^ ^]^ and cationic lipid‐modified aminoglycosides (CLAs)^[^ ^94^ ^]^ in LNPs can also facilitate the endosomal escape of mRNA. These studies revealed composition‐optimized LNPs hold great potential in overcoming the bottlenecks of mRNA delivery.

However, synthetic cationic lipids with permanent positive charge may cause harmful side effects to cells.^[85c]^ To address the cytotoxicity of cationic lipids, ionizable lipids (such as DLinDMA, DLin‐MC3‐DMA) were developed to expand the therapeutic window of LNP.^[^ ^95^ ^]^ Furthermore, researchers added ester or amide bonds in the lipid tails to endow ionizable lipids with biodegradable properties for reduced toxicity (such as L319).^[^ ^96^ ^]^ For instance, ionizable lipids with STING pathway activity were recently excavated to enhance the immune activation efficiency of mRNA‐LNP.^[^ ^72^, ^76^ ^]^ Bogaert et al. added tricyclic cationic amphiphilic drugs (CADs) into LNP, which behaved both as structural components of LNP and pharmacological molecules.^[^ ^97^ ^]^ The constructed mRNA‐LNP, namely CADosomes, showed a synergic antitumor effect of CADs and mRNA with decreased cytotoxicity than cationic lipids. In the future, increased efforts are highly desired to design novel lipids with additional immune‐activating functions for LNP‐based mRNA delivery and cancer immunotherapy.

Several points need to be considered when utilizing LNP to deliver mRNA in vivo apart from optimizing the compositions of LNPs. First, reports showed that the cellular responses to LNPs vary significantly in different species.^[^ ^98^ ^]^ This indicates the transfection efficacy of mRNA‐loaded LNP may differ in experimental animal models and humans. Dobrowolski et al. constructed a single‐cell NP targeting‐sequencing (SENT‐seq) technology, which could precisely investigate the distribution of LNPs with distinct structures. They demonstrated that cell heterogeneity plays a crucial role in the in vivo behavior of mRNA‐LNPs with different compositions.^[^ ^98^ ^]^ Second, Paunovska et al. found that increased phosphatidylinositol (3,4,5)‐triphosphate (PIP3) activity led to limited LNP mRNA delivery efficiency due to excessive consumption of cellular resources, rather than cell uptake or endosomal escape.^[^ ^99^ ^]^ This suggests the metabolic state of cells may affect mRNA‐delivering efficiency by LNPs. Third, the on‐and‐off of inflammatory signaling in the target cells may play a role in the mRNA translating process. Lokugamage et al. discovered that activation of TLR4 inhibited mRNA translation in tested cell types, suggesting that the inflammatory state of cells plays a role in mRNA translation.^[^ ^100^ ^]^ In general, species and cell types, the metabolic and inflammatory states of cells should be emphasized in investigating targeted delivery of mRNA with LNPs for cancer immunotherapy.

### 3.2 Gel‐like materials for mRNA delivery

Gel‐like materials were also exploited for mRNA delivery for prolonged drug release and immune response.^[^ ^101^ ^]^ Yin et al. reported an injectable hydrogel composed of GO and polyethylenimine for preparing an antitumor mRNA platform. The constructed system can enhance the stability of mRNA and accumulate in lymph nodes (LNs) specifically. Results showed that the hydrogel could release mRNA continually for at least 30 days and significantly increase the number of antigen‐specific CD8^+^ T cells to inhibit tumor growth (Figure 3B,C).^[^ ^66a^ ^]^

Furthermore, gel‐based systems possess high drug loading capacity, providing the opportunity for combinational therapy of mRNA and other treatments. Dastmalchi et al. developed a hydrogel‐based platform loaded with CXCL9 for DC‐targeted mRNA delivery.^[^ ^102^ ^]^ CXCL9 is employed to chemically attract activated B cells, monocytes, CD8^+^ T cells, and CD4^+^ Th1 T cells. The CXCL9 and mRNA co‐loaded hydrogel synergistically induced antitumor response and improved survival of murine glioblastoma (GBM)‐bearing mice with a single dose, revealing the co‐loading superiority of hydrogel‐based platforms.

### 3.3 Polymers‐based platforms for mRNA delivery

Polymer‐based delivery platforms are also widely used in mRNA tumor therapy.^[^ ^105^ ^]^ Biodegradable and biocompatible polymers can encapsulate and precisely deliver various antigens or mRNAs to DCs, where their uptake by DCs is leveraged. Polyethylenimine can be used for preparing polymeric micelle to deliver mRNA. As a paradigm, Ren et al. modified polyethylenimine with vitamin E succinate and obtained an mRNA vehicle with low cytotoxicity and a high mRNA transfection rate.^[^ ^106^ ^]^ Tan et al. conjugated branched polyethylenimine with β‐cyclodextrin to form a polymer‐based NP for mRNA delivery and induced a potent immune response, which possesses excellent potential in anti‐tumor therapy.^[^ ^32^ ^]^ Polymers can also be combined with conventional LNP formations for mRNA delivery. Persano et al. used a cationic poly‐(β‐amino ester) (PBAE) to condense mRNA into a stable polyplex NP via electrostatic interaction, which was subsequently encapsulated into a classical LNP shell.^[^ ^73^ ^]^ The hybrid platform displayed an intrinsic adjuvant activity through TLR7/8 signaling and reduced over 90% of tumor nodules in lung metastatic melanoma‐bearing mice. In a related study, Kaczmarek et al. also used PBAE to deliver mRNA and DNA and reached a lung endothelium‐targeting effect after intravenous injection in mice.^[^ ^107^ ^]^

However, negatively or neutrally charged polymers inhibit the combination of polymer and mRNA,^[^ ^108^ ^]^ which needs a combination of polymer with other cationic materials. To address the above challenges, Coolen et al. chose cationic cell‐penetrating peptides (CPPs) as a bridge to link mRNA and polymer.^[^ ^103^ ^]^ CPPs are a kind of membrane‐active peptide that can disrupt membranes for endosomal release, facilitating cytosol delivery of mRNA.^[^ ^109^ ^]^ As a result, they constructed PLA‐NPs to vectorize mRNA and efficiently transport mRNA to DCs to trigger PRRs activation and potentiate innate immune response (Figure 3D).

Additionally, the structure of the polymer is crucial for the targeting ability and endosomal disruption efficiency of the delivery system.^[^ ^110^ ^]^ Yu et al. demonstrated that chemical modification of polyesters by changing alkyl chain length and molar ratio in the formulation can adjust the delivery selectivity between organs of polymer‐based platforms.^[^ ^111^ ^]^

### 3.4 Peptide‐based platforms for mRNA delivery

Peptide‐based delivery systems have gained impressive attention in mRNA delivery.^[^ ^103^, ^112^ ^]^ CPPs were employed to facilitate cellular uptake of mRNA by assembling negatively charged glycosaminoglycans on the cell surface, thus inducing micropinocytosis.^[^ ^113^ ^]^ Udhayakumar et al. described CPPs containing the arginine‐rich amphipathic RALA motif, which can condense mRNA into nanocomplexes and deliver mRNA to DCs.^[^ ^109^ ^]^ Also, RALA mRNA nanocomplexes can disrupt membranes in an acid‐dependent manner, which ensures the high endosomal escape and protein expression rate of mRNA and subsequently elicits robust antigen‐specific T‐cell responses in vivo. Kim et al. designed an amphipathic CPP/mRNA complex with optimal charges by adjusting the amine/phosphate ratio, which showed impressive mRNA protection against RNase, improved cellular uptake and protein expression.^[^ ^114^ ^]^ Other peptide‐based biomaterials like α‐helical cationic peptide “KALA” were also used in mRNA delivery.^[^ ^115^ ^]^ To compensate for the low endosomal escape rate of mRNA, Sun et al. developed pH and redox‐sensitive coacervate microdroplets by liquid‐liquid phase separation to transport macromolecular therapeutics into cells directly, bypassing classical endocytic pathways (Figure 3E).^[^ ^104^ ^]^ After cytosol release, the coacervates undergo glutathione‐mediated release of mRNAs and exhibit a high transfection efficiency and protein expression level. This peptide coacervate strategy overcomes the general limitation of endosomal escape fundamentally, representing a promising formulation for intracellular delivery of mRNA to treat cancer.

Xenopeptides are sequence‐defined peptide‐like macromolecules, where artificial amino acids replace natural amino acids. In a bioinspired chemical evolution strategy, amphiphilic xenopeptides were screened for RNA delivery.^[^ ^116^ ^]^ Lipo‐xenopeptides were generated by solid phase‐assisted synthesis combining natural amino acids with artificial aminoethylene amino acids such as succinoyl tetraethylene pentamine (Stp) and natural or artificial fatty acids. Both the sequence and topology of these carriers strongly influenced the stability and biological activity of the formed RNA complexes (‘polyplexes’). Chemical evolution revealed that a careful balance between polyplex stabilization by lipidic residues and sufficient cargo release within the transfected cell is required. For mRNA delivery, the incorporation of a bioreducible disulfide bond between the cationic backbone and the lipidic side chain of the carrier resulted in effective mRNA release in the cytosolic reductive environment.^[^ ^116a^ ^]^ Alternatively, the incorporation of lipo amino fatty acids (LAFs) resulted in double pH‐responsive mRNA carriers with high potency for endosomal escape and in vivo activity upon systemic application in mice.^[^ ^116b^ ^]^ Screening lipo‐xenopeptides for genome editing using Cas9 protein/single guide RNA (sgRNA) ribonucleoprotein (RNP) polyplexes, Lächelt and colleagues observed that hydroxystearic acid (OHSteA) was far superior to stearic acid as lipidic carrier component^[^ ^116c^ ^]^. Incorporation of folic acid (FolA)‐PEG for receptor‐mediated uptake improved gene editing of receptor‐positive carcinoma in vitro and in vivo.^[^ ^116d^ ^]^ Targeting two immune checkpoint genes, PD‐L1 and PVR, by injection into CT26 colon cancer in vivo induced CD8^+^ T cell recruitment and distinct CT26 tumor growth inhibition^[^ ^116d^ ^]^ Systematic variation of the number and types of artificial oligoamino acids and applied fatty acids of the xenopeptide sequences revealed a relationship between the logD_7.4_ and Cas9/sgRNA RNP‐mediated genome editing potency. The highly potent carrier TFE‐IDAtp1‐LinA contained a trifluoroethyl‐iminodiacetic acid analog of Stp, linoleic acid as fatty acid residue, and achieved target gene knockout with a 50% effective concentration EC_50_ of 0.38 nm RNP.^[^ ^116e^ ^]^

Other viral and cell‐based vehicles like biomimetic polymers,^[^ ^65^, ^117^ ^]^ exosomes,^[^ ^118^ ^]^ microbial cell wall‐derived polysaccharides,^[^ ^77^ ^]^ and extracted CM are booming in the mRNA delivery field because of their superior biocompatibility, biodegradability, and intrinsic targeting ability.^[^ ^113^, ^119^ ^]^ Park et al. expressed the virus hemagglutinin protein on CM by genetic engineering approaches.^[^ ^120^ ^]^ The constructed virus‐mimicking CM was subsequently coated on mRNA‐loaded NPs. As a result, the hemagglutinin facilitated mRNA release into cytoplasm at endosomal pH values. The study offered a novel biosynthetic strategy for constructing biomimicking mRNA delivery systems with superior endosomal escape efficiency and is expected to facilitate clinical application of mRNA vaccines.

## 4 TARGETED MRNA DELIVERY FOR POTENTIATED CANCER IMMUNOTHERAPY

mRNA‐based immunotherapy aims to harness proteins produced from delivered mRNA in host cells to induce efficient immune response and is promising for substituting protein replacement therapy. Since most of the missing or abnormal proteins are produced in specific cells of organs, it is required to deliver mRNA selectively to these aiming sites, representing the idea of precise medicine. Targeted delivery of mRNA can effectively reduce the off‐target and side effects of drugs and maximize drug efficacy. Currently, the drug delivery system of non‐viral NPs allows for repeated administration, and LNPs represent the most widely used vectors. LNPs are versatile delivery vehicles with tunable physicochemical properties ideally suited for vaccine delivery and mRNA therapeutics.^[^ ^17^, ^121^ ^]^ In addition, the two mRNA vaccines approved by the FDA for clinical prevention of novel coronavirus infection adopted LNP platforms.^[^ ^11d,e,g^ ^]^ It is important to regulate the in vivo distribution behavior of LNP while retaining its existing merits by adding extra agents with an affinity for specific organs or cells. In cancer treatment especially, developing a platform for targeted delivery of mRNA is crucial for triggering a powerful immunotherapy effect to kill tumor cells.^[^ ^12^ ^]^

### 4.1 Organ‐specific mRNA delivery

Organ‐specific mRNA delivery systems are designed to selectively treat lesions in specific organs, tissues, or cell types. Targeted drugs can effectively maximize therapeutic efficacy and decrease toxic and side effects on unrelated tissues or organs.^[^ ^122^ ^]^ LNPs are known for their highly effective RNA delivery to liver hepatocytes. For example, Onpattro, an LNP formulation of siRNA, which was approved by FDA for treating polyneuropathies in 2018, delivers siRNA to hepatocytes with high potency.^[^ ^123^ ^]^ To translate the clinically approved delivery platform to mRNA‐based therapy, Wang et al. used liver‐homing MC3 LNPs to selectively deliver mRNA to the liver with a high transfection efficacy while drastically less in other organs.^[^ ^124^ ^]^ Similarly, Rybakova et al. used liver‐targeting LNPs to deliver modified mRNA encoding an anti‐human epidermal growth factor receptor 2 (HER2) antibody, trastuzumab, into the liver which reached the expression of full‐size therapeutic antibodies to elicit potent antitumor effect.^[^ ^125^ ^]^ Apart from targeting hepatocytes, transporting mRNA to liver microenvironmental cells represents an attractive strategy for treating liver cancer. Paunovska et al. formulated LNP with oxidized cholesterol which preferentially delivered mRNA into liver microenvironmental cells (e.g., liver endothelial cells, Kupffer cells), with a five‐fold transfection rate than hepatocytes.^[^ ^126^ ^]^ These liver‐targeting mRNA‐delivering platforms hold great potential in hepatoma immunotherapy.

Given that most of the systemically administrated mRNA delivery systems are intended to accumulate in the liver,^[^ ^58^, ^86^, ^124^, ^127^ ^]^ transporting mRNA outside the liver is urgently needed for certain extrahepatic diseases, such as extrahepatic cancers.^[^ ^128^ ^]^ By varying the lipid‐to‐mRNA weight ratio and subsequently adjusting the surface charges of LNP, Kranz et al. reported a pioneering study for surface‐charge dependent organ tropism of mRNA‐LNP, opening the prelude to using LNP to deliver mRNA in vivo.^[^ ^129^ ^]^ Based on the well‐known LNP compositions, they precisely delivered mRNA‐encoding mutant neo‐antigens to DCs in vivo by optimally adjusting the net charges of LNPs. The transported mRNA can efficiently express the targeted antigens, thus inducing potent memory T‐cell responses for cancer immunotherapy in B16‐OVA lung metastasis models.^[^ ^129^ ^]^

**FIGURE 4:**
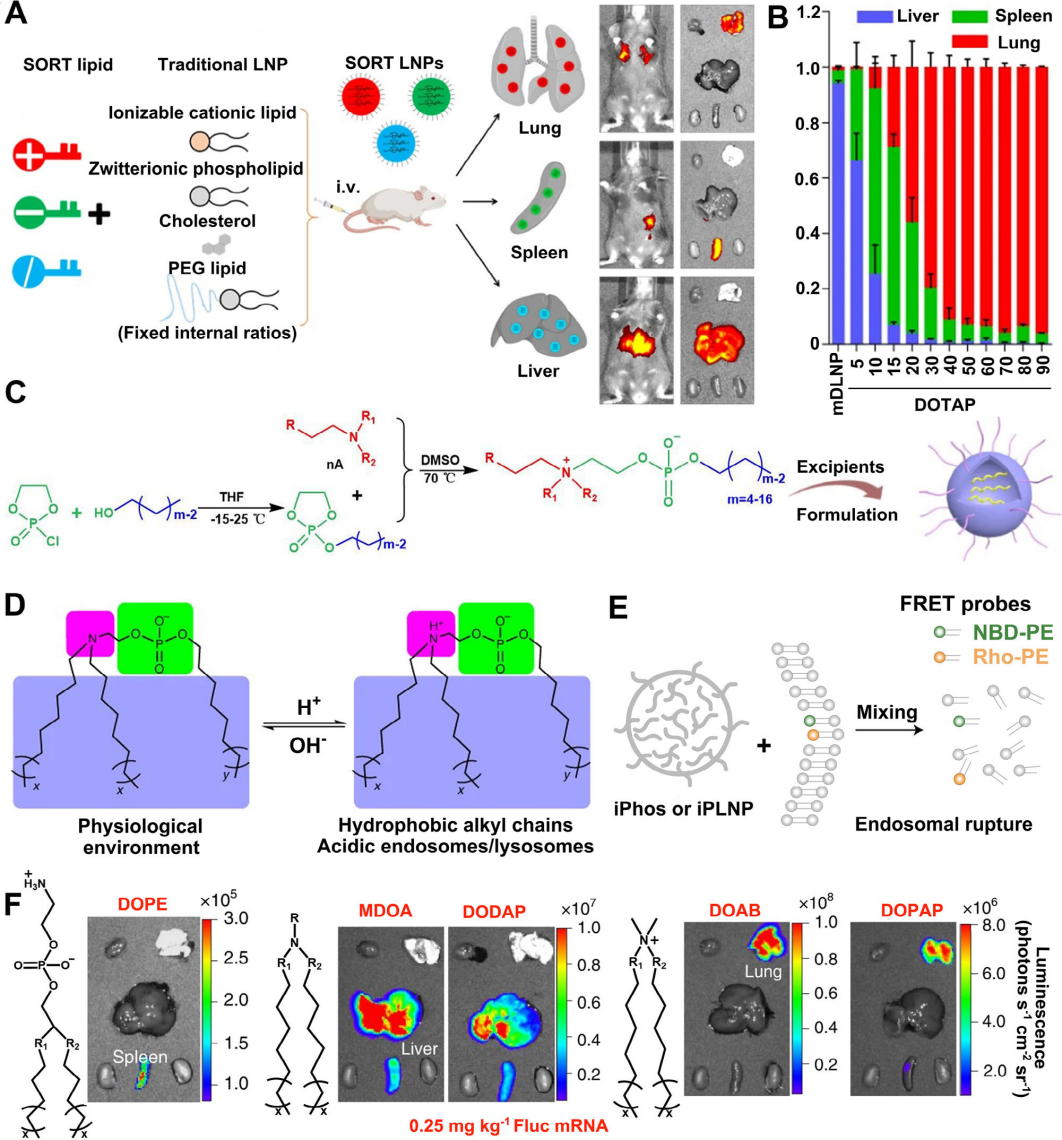
The addition of functionalized lipids for organ‐specific mRNA delivery. A Organ‐specific delivery of mRNA by adding SORT lipids to traditional LNPs. B The percentage of SORT molecule in LNP in altering the tissue‐specific delivery of mRNA. Reproduced with permission.^[^ ^123^ ^]^ Copyright 2020, Nature Publishing Group. C The synthetic routes of iPhos and iPLNP. D The structure of iPhos lipids. E Schematic representation for lipid fusion, membrane rupture, and iPLNP dissociation. F Images of fluorescence in spleen, liver or lung by iPLNPs containing zwitterionic, ionizable cationic, and permanently cationic helper lipids, respectively. Reproduced with permission.^[^ ^131^ ^]^ Copyright 2021, Nature Publishing Group.

Based on the speculation that the internal or external charges of LNPs can modulate their tissue‐targeting ability, Cheng et al. reported organ‐specific delivery of mRNA by adding internal charge‐tuning lipids (zwitterions lipids, ionizable lipids, cationic lipids, anionic lipids, etc.), termed selective organ targeting (SORT) molecules, into the LNPs to alter their in vivo distribution profile.^[^ ^54^ ^]^ To be specific, LNPs with permanently cationic SORT lipids (DDAB, EPC) accumulate preferentially in the lung, LNPs with anionic SORT lipids (14PA, 18BMP) accumulate in the spleen, and LNPs with ionizable cationic SORT lipids with tertiary amino groups (DODAP, C12‐200) accumulate to the liver. In mechanism, the SORT lipids recognize and bind to specific plasma proteins after desorption of PEGylated lipids on the surface of LNPs. Subsequently, LNPs target different organs through the interaction of adsorbed proteins with homologous receptors highly expressed in particular tissue.^[^ ^130^ ^]^ As a result, the SORT‐added LNPs achieved targeted mRNA delivery to the lung, spleen, and liver, respectively, and the efficient production of therapeutic‐level proteins including human hemoglobin and mouse interleukin (Figure 4A). The percentage of SORT molecules in LNPs also extensively altered the tissue‐targeting ability of mRNA (Figure 4B). The SORT molecules in the study overcame the hepatocyte accumulation challenges of LNPs and are expected to promote protein replacement therapy of cancer.^[^ ^123^ ^]^

Similarly, by changing a single lipid in the compositions of Onpattr, Pattipeiluhu et al. altered the surface charge of LNP from neutral to anionic.^[^ ^132^ ^]^ This charge conversion significantly enhanced the hepatic reticuloendothelial system‐targeting delivery of mRNA, suggesting the surface charge plays a critical role in the targeting behavior of LNP. Although a report showed that hydrogen‐bond interaction, ionization, and proportion of ionizable lipids are crucial for the biodistribution of mRNA‐LNP,^[^ ^121^ ^]^ the mechanisms behind the tissue tropism and internal or surface charges of LNP still need further investigation.

Another challenge of mRNA therapeutics lies in improving the protein expression levels of mRNA, which needs highly efficient delivery systems.^[^ ^133^ ^]^ Endosomal escape is of great importance in mRNA delivery process. Inspired by the design idea of cationic lipids (using ionizable amines and multiple alkyl chains to acquire charges for endosomal escape), Liu et al. integrated the advantages of cationic lipids into phospholipids.^[^ ^131^ ^]^ They designed ionizable phospholipids (iPhos) with membrane integration potential, which were composed of one tertiary amine, one phosphate group, and three alkyl tails (Figure 4C). The pH‐switchable zwitterionic heads and multiple tails of iPhos facilitate its insertion into the endosomal membrane and induce a hexagonal phase transition, which significantly enhances the efficiency of mRNA endosomal escape (Figure 4D,E). More importantly, organ‐targeting ability can be imparted to delivery systems by modulating the chain length of iPho lipids. Using iPho lipids, zwitterions, and helper lipids, they prepared an LNP delivery system that could selectively deliver mRNA to the spleen, liver, or lung via intravenous administration (Figure 4F). The synthesized ionizable phospholipids endow LNPs with superiorities of mRNA organ‐targeted delivery to different organs, which is expected to achieve effective immunotherapy of tumors in various tissues.

To further expand the material space of LNPs, many researchers explored the possibilities of adjusting the compositions of LNPs for organ‐selective mRNA delivery. For example, Zhang et al. added helper lipid 1,2‐distearoyl‐sn‐glycero‐3‐phosphocholine (DSPC) into LNP to deliver mRNA and found that this LNP preferentially accumulated in the spleen and liver in an ApoE‐dependent manner.^[^ ^86c^ ^]^ Kong et al. further demonstrated that LNP formulated with thiolated‐DSPE (termed LNPs‐SH) could bind with the cysteine domains of the bladder via a disulfide bond.^[^ ^134^ ^]^ As a result, LNPs‐SH successfully adhered to the bladder and continually delivered mRNA encoding lysine‐specific demethylase 6A (KDM6A, a histone demethylase) for bladder cancer therapy. Miao et al. obtained new lipids by introducing alkynes and ester groups into the lipid tails of Dlin‐MC3‐DMA, and co‐formulated LNP with other materials containing amine structures to achieve efficient delivery of mRNA.^[^ ^127a^ ^]^ The albumin modification of the LNP surface by co‐incubating with serum significantly promoted cellular uptake of LNP through the ApoE‐independent pathway in the liver. Meanwhile, adding alkyne lipids increased the endosomal membrane fusion of LNPs to facilitate mRNA release to the cytoplasm.

**FIGURE 5:**
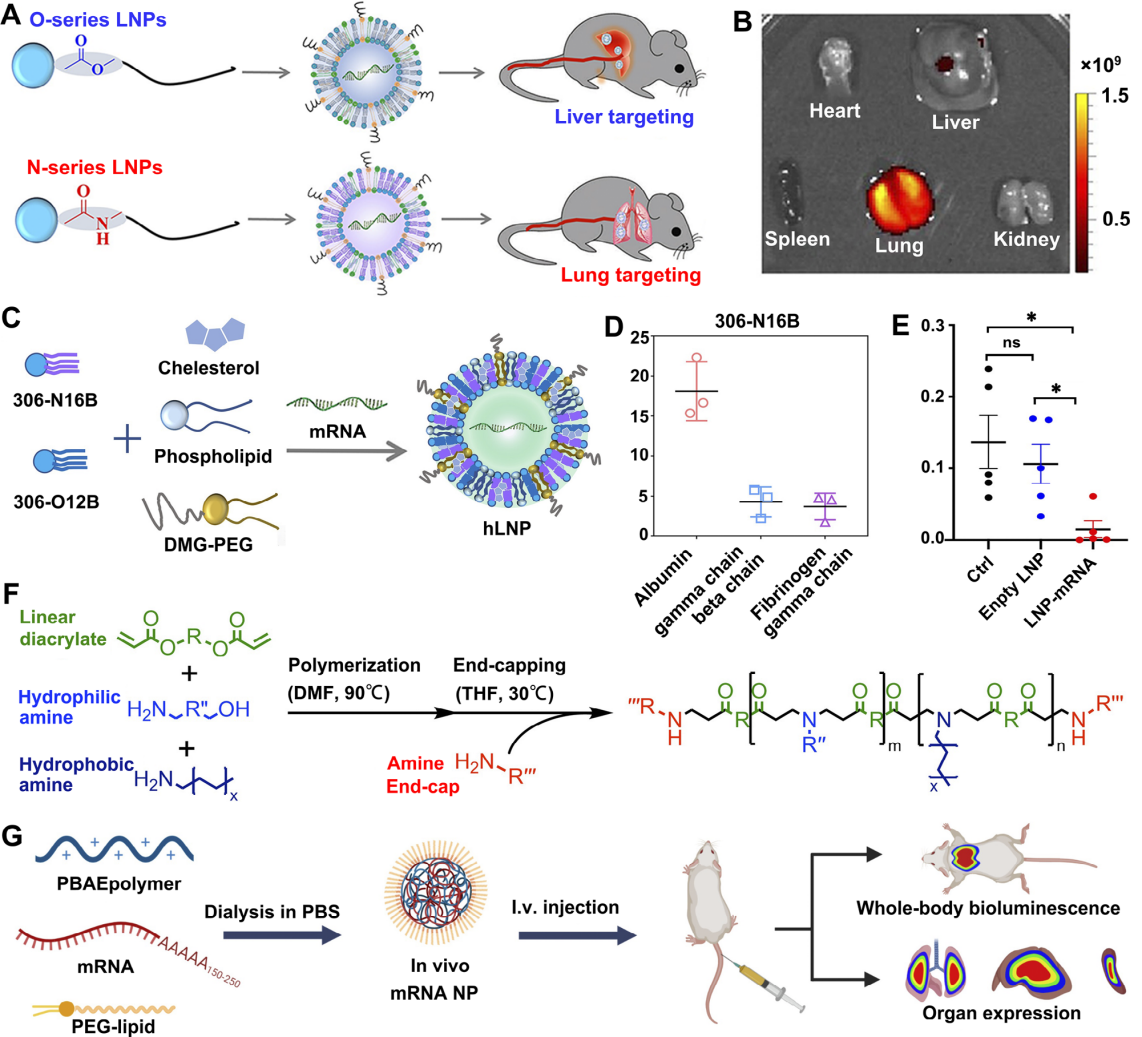
Schematic illustration of adding synthetic N‐series lipids and polymer in NPs for organ‐targeted mRNA delivery. A O‐ and N‐series lipid‐based LNPs with liver and lung targeting ability, respectively. B N‐series lipid 306‐N16B‐based LNP‐mediated preferential transport of mRNA to the lungs. C The preparation process of hLNP with a mixture of 306‐O12B and 306‐N16B lipid. D Percentage of proteins in the protein corona absorbed on lung‐targeting LNP. E Fraction of tumor nodes treated with Tsc2 mRNA‐loaded hLNP and control group. Reproduced with permission.^[^ ^90^ ^]^ Copyright 2022, National Academy of Sciences. F The synthetic route of PBAE, a linear end‐capped polymer. G The preparation process of PEG‐coated mRNA NPs containing PBAE polymers. Reproduced with permission.^[^ ^110^ ^]^ Copyright 2022, American Association for the Advancement of Science.

In addition to modified known lipids, new synthetic lipids can also alter the biodistribution of mRNA‐LNPs. Li et al. developed cholesteryl‐based disulfide bond‐containing biodegradable cationic lipidoid NPs for mRNA delivery to the lung and spleen via intravenous injection.^[^ ^135^ ^]^ Qiu et al. synthesized a library of lipidoids with verified tail structures and found that O‐series lipidoids (with an ester bond in the tails) are prone to deliver mRNA into the liver,^[^ ^136^ ^]^ while N‐series lipidoids (with an amide bond in the tails) tend to transport mRNA to the lungs following systemic administration (Figure 5A,B).^[^ ^90^ ^]^ It is found that the N series lipidoid 306‐N16B‐based LNP tends to absorb a layer of serum proteins (e.g., serum albumin, fibrinogen beta chain, fibrinogen gamma chain) to form protein corona, which serves as target ligands to orient LNP to the specific organ (Figure 5D). They also tested the therapeutic efficacy of this platform in pulmonary lymphangioleiomyomatosis (LAM). They constructed a hybrid LNP (hLNP) formulated with synthetic lipids, 306‐N16B and 306‐O12B, for delivering mRNA encoding tuberous sclerosis complex 2 (Tsc2, whose inactivating mutations can cause pulmonary LAM) (Figure 5C) to significantly suppress tumor growth in TTJ (kidney‐derived epithelial tumor cells) tumor‐bearing mice (Figure 5E).^[^ ^90^ ^]^

Polymer‐based NPs can also achieve organ‐specific delivery of mRNA. Rui et al. demonstrated that polymer structure altered the organ selectivity of polymer NPs for mRNA delivery in vivo.^[^ ^110^ ^]^ They first synthesized biodegradable PBAE with verified backbone hydrophobicity and terminal structure and explored the relationship between the polymer structure and the function of the formed NPs to deliver mRNA (Figure 5F,G). The results showed that increasing backbone hydrophobicity increased mRNA expression in all evaluated organs while altering the polymer end groups enabled targeted delivery of mRNA to the lungs and liver. This suggests that PBAE‐based NP is a promising platform for enhanced organ‐selective delivery of mRNA. Palmiero et al. also synthesized biodegradable PBAE carriers and delivered mRNA to the spleen selectively by adjusting the caprolactone units on PBAE through intravenous injection.^[^ ^105b^ ^]^

By modulating the hydrophobicity of functional polyesters, Yu et al. synthesized an optimal polymeric carrier for the targeted transportation of mRNA to the lungs and spleen.^[^ ^111^ ^]^ Based on the fact that cell membranes also contain amphiphilic lipids, they verified that the hydrophobicity plays an essential role in the targeting ability of the polymer whose cellular uptake is dominated by clathrin‐dependent endocytosis.

Ionizable polymers can also be used for tissue‐selective mRNA delivery with improved endosomal escape of mRNA. Kowalski et al. constructed LNPs with a series of synthetic ionizable amino‐polyesters (APEs), which preferentially locate and elicit efficient mRNA expression in specific organs (e.g., lung, liver, spleen, etc.).^[^ ^137^ ^]^ For instance, Zhang et al. co‐assembled mRNA with the newly synthesized ionizable amphiphilic Janus dendrimer (IAJD) to efficiently deliver mRNA to the lungs in vivo.^[^ ^138^ ^]^ Changing the hydrophilic groups and replacing amide of IAJDs with ester groups, altered the delivery of mRNA from lung to spleen or liver. Besides, Liu et al. synthesized a series of phospholipid‐modified zwitterionic phospholipidated polymers (ZPPs), which delivered mRNA preferentially to the spleen and LNs.^[^ ^139^ ^]^ The zwitterionic property enhances the serum resistance of the polymer‐based system, and side alkyl chains can improve the endosomal escape of mRNA The research expanded the applications of polymer‐based mRNA therapeutics. However, the relationship between in vivo mRNA distribution and polymer modification with side alkyl chains needs further investigation.^[^ ^111^ ^]^

Polymers can also be combined with lipids for in vivo mRNA delivery. For example, Yang et al. designed a hybrid NP composed of a PLGA‐core and lipid‐shell for the co‐loading of adjuvant gardiquimod and mRNA, respectively.^[^ ^64^ ^]^ The intravenous administration of the hybrid NP induced enriched mRNA expression in the spleen and a robust immune response for tumor inhibition in melanoma tumor‐bearing mice.

**FIGURE 6:**
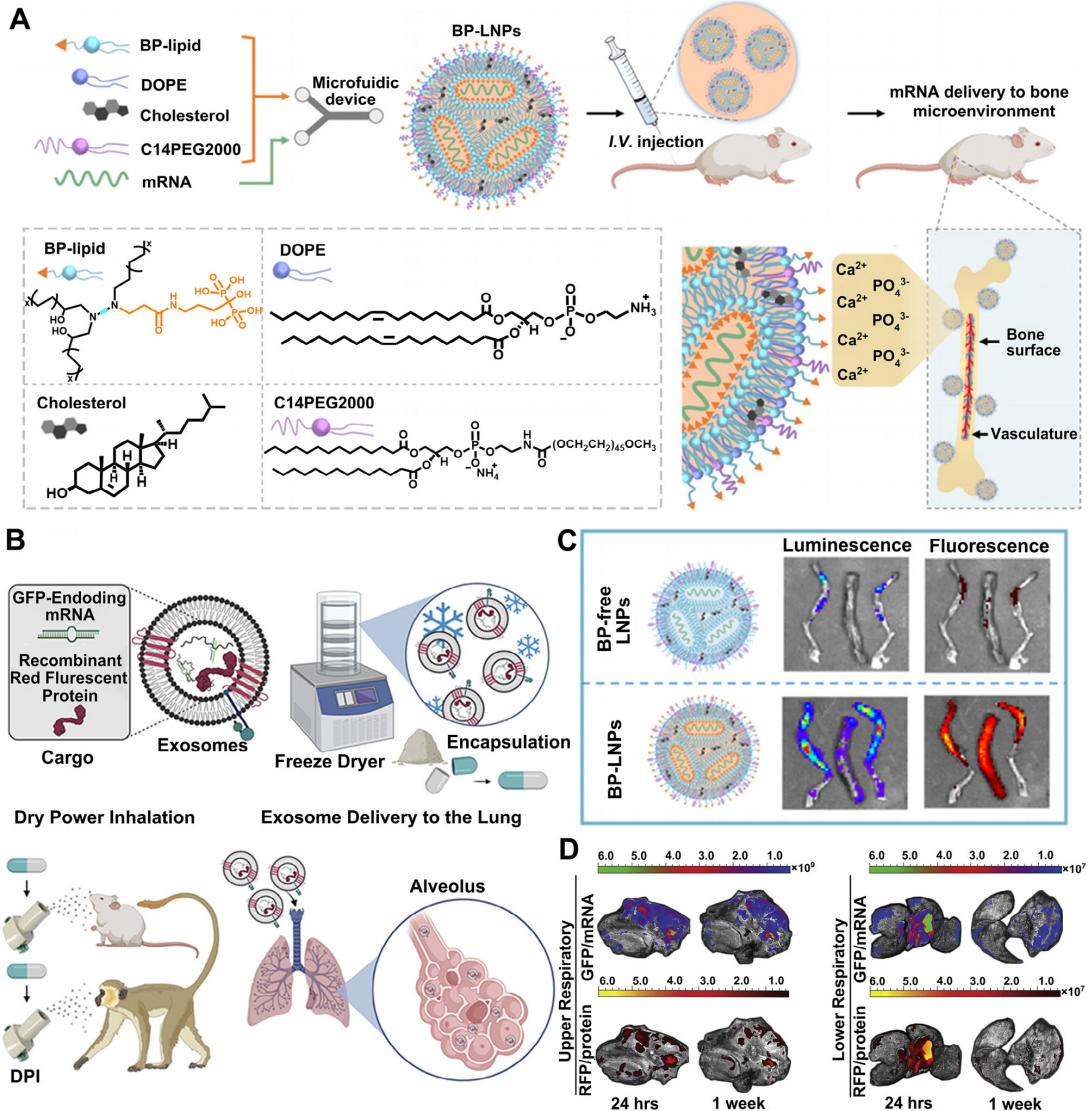
The application of bisphosphonate lipid and Lung‐Exos for bone‐ and lung‐targeted delivery of mRNA respectively. A The scheme of delivering mRNA to bone microenvironment in vivo with BP‐based LNP via coordination of BP with Ca^2+^. B The addition of BP in LNP significantly increased the distribution of mRNA cargos in the bone microenvironment (left to right: left leg, spine, and right leg). Reproduced with permission.^[^ ^141^ ^]^ Copyright 2022, American Chemical Society. C Preparation and dry powder inhaling administration of mRNA‐loaded Lung‐Exos. D Distribution of mRNA‐loaded Lung‐Exos in the respiratory tracts via DPI. Reproduced with permission.^[^ ^145^ ^]^ Copyright 2022, Elsevier.

Lipid‐like materials with unique properties can also be used for the targeted delivery of mRNA to the bone microenvironment, which is necessary for treating bone‐related diseases such as osteoarthritis, osteomyelitis, and bone cancer.^[^ ^140^ ^]^ To overcome the biological barriers (such as low blood flow and low affinity between drugs and bone minerals) of transporting mRNA into the bone microenvironment, Xue et al. designed a series of bisphosphonate lipid‐like materials and combined them with three other conventional compositions to form an LNP platform (Figure 6A).^[^ ^141^ ^]^ After systemic administration, the best‐performing BP, 490BP‐C14, which possesses a satisfying affinity for bone minerals like calcium ions (Ca^2+^), successfully transported mRNA‐encoding bone morphogenetic protein‐2 (BMP‐2) to the bone microenvironment and elicited protein expression for bone development (Figure 6A,B). Additionally, Badieyan et al. used collagen sponges to deliver mRNA encoding human BMP‐2 into the bone microenvironment and achieved sustained mRNA release for bone regeneration.^[^ ^142^ ^]^ The study emphasized the eminent property of collagen sponges in drug delivery and the promising future of mRNA in regenerative medicine.

Optimizing administration routes can also achieve the organ‐selective delivery of mRNA. To repair the functional damaged tissue in myocardial infarction, Labonia et al. locally administrated LNPs‐mRNA with adjusted type or amount of helper lipids to the left ventricular wall and achieved improved cellular tropism of mRNA delivery.^[^ ^143^ ^]^ Similar work by Evers et al. also demonstrated the feasibility of transporting mRNA to the infarct region after myocardial infarction with LNPs.^[^ ^144^ ^]^ However, the expression level of mRNA was still high in other organs like the liver and lungs in both studies, suggesting that the myocardium‐targeted delivery of mRNA still needs improvement. Pulmonary drug delivery methods (such as nebulization and inhalation), which deliver therapeutics into the vascularized and bronchial pulmonary alveoli via local, non‐invasive and absorptive inhaling administration, have been applied in mRNA‐based therapy for respiratory diseases. Popowski et al. took lung‐derived extracellular vesicles (EVs) or exosomes (Lung‐Exos) as the carrier of mRNA, formulated as a lyophilized powder and packed into capsules to enhance its room‐temperature stability (Figure 6C).^[^ ^145^ ^]^

Compared with the commercial liposome standard, Lung‐Exos successfully delivered mRNA to the bronchioles and parenchyma of lungs by dry powder inhalation (DPI) (Figure 6D). Similarly, Qiu et al. delivered mRNA to deep lung regions by dry powder formulation of PEG‐modified KL4 protein through intratracheal administration and achieved superior transfection efficiency in mouse lungs.^[^ ^146^ ^]^ Lokugamage et al. optimized the composition of LNPs made of lipids, helper lipids, and PEG through a cluster approach and then successfully delivered mRNA encoding neutralizing antibodies against hemagglutinin to lungs by nebulization.^[^ ^86a^ ^]^ It was more effective in protecting mice from the H1N1 subtype of influenza virus than intravenous administration. The study demonstrated the critical role of PEG ratio in LNP performance and that aerosolized mRNA delivery is very suitable for treating lung diseases, especially lung infection and lung cancer. However, the relationship between the aerosolized delivery effect and LNP components still needs further exploration.

Biodegradable polymers represent another optimal material for lung‐targeted delivery of mRNA via inhalation administration. Patel et al. synthesized hyperbranched PBAE to form polyplexes with mRNA, which were distributed in lung epithelial cells throughout all five lobes and reached high protein expression after aerosol inhalation without transfecting other tissues.^[^ ^147^ ^]^ These reports emphasized the potential of mRNA‐based inhalable formulations in respiratory disease treatment. Pulmonary transportation of mRNA can also be achieved by intravenous administration. Anderson's group combined PBAE with PEG‐lipid to form a hybrid‐LNP, delivering mRNA intravenously into lung endothelium and pulmonary immune cells in mice.^[^ ^148^ ^]^ The formulation achieved potent systemic delivery of mRNA to the lungs and efficient protein expression in pulmonary immune cells, representing a promising approach for treating pulmonary disease.

Generally, organ‐specific delivery of mRNA can be achieved through adding internal or external charge‐tuning lipids, new synthetic functional lipids into LNP formulation, adjusting the structure of polymers, designing lipid‐like materials with unique properties, etc. Despite the study of targeted mRNA delivery in the laboratory having reached the cell‐targeting level, the clinical application of organ‐specific delivery is still faced with significant challenges that need further investigation.

### 4.2 Tumor cell‐targeted mRNA delivery

Selectively transporting mRNA to tumor cells to express cytotoxic proteins and proteins that are under‐expressed in tumor cells (e.g., tumor suppressor proteins, cytokines, tumor‐associated antigens [TAAs]) is a promising method of cancer therapy.^[^ ^7^, ^58^, ^149^ ^]^ These proteins favor recognizing and presenting antigens, restoring the functions of tumor suppressor genes in tumor cells to inhibit tumor growth by regulating the level of specific cytokines and reversing the ITM.^[^ ^10^, ^50^, ^150^ ^]^ In this strategy, the targeted delivery of mRNA to the cytoplasm of tumor cells is particularly critical, which is an essential prerequisite to achieve the high‐efficiency expression of target proteins in tumor cells and to reduce the toxic side effects to normal cells with the least amount of mRNA.

**FIGURE 7:**
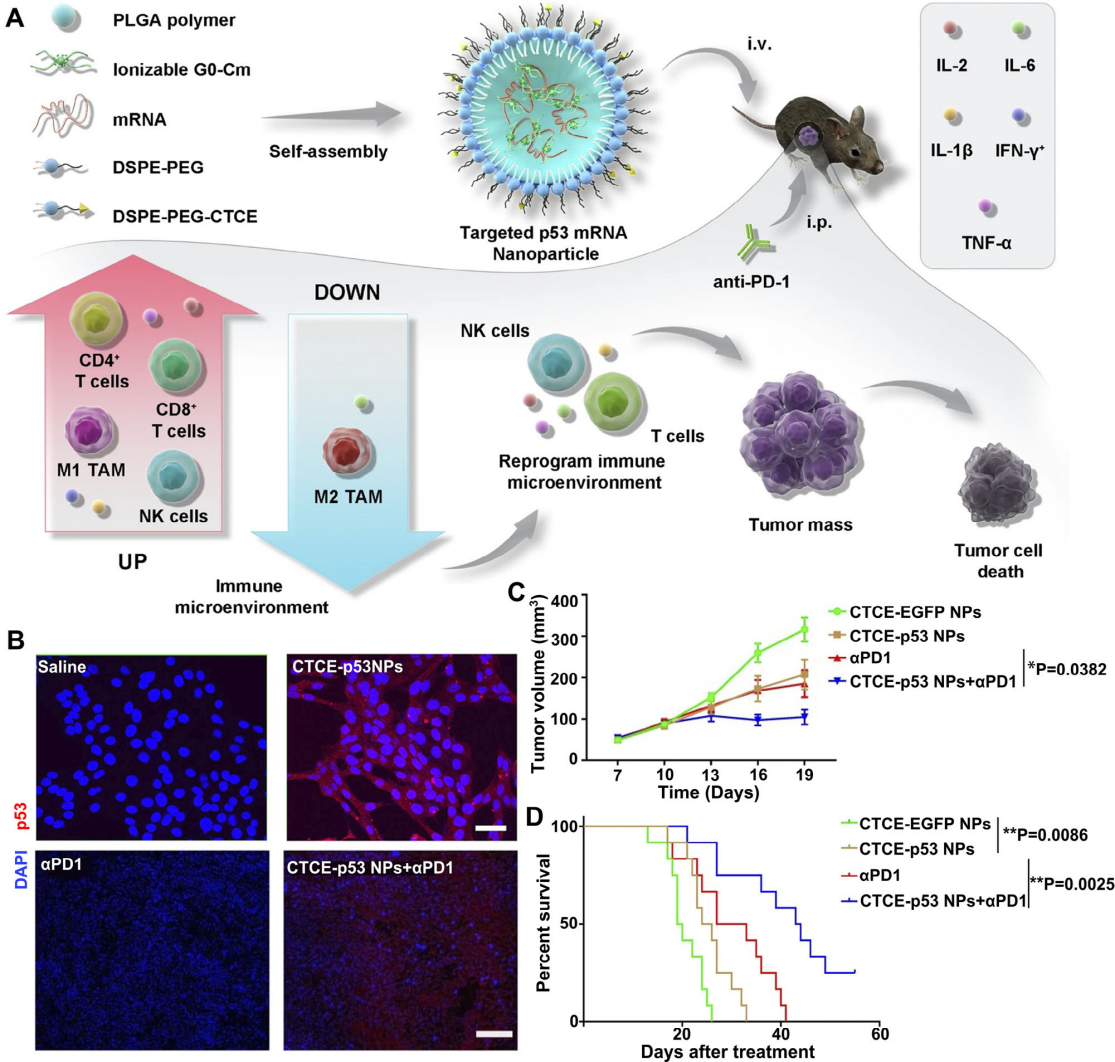
Schematic diagram of surface‐modified NPs with protein for tumor cell target delivery of mRNA. A Delivering CXCR4‐targeted p53 mRNA and anti‐PD‐1 NPs to p53‐deficient murine HCC cells (RIL‐175) for enhanced immune response. B Immunofluorescence for p53 (red signals) in RIL‐175 cells after treatment with CTCE‐p53 NPs and other groups. C,D Tumor growth rate and survival curves of different treatment groups in RIL‐175 orthotopic mouse model. Reproduced with permission.^[^ ^152^ ^]^ Copyright 2022, Nature Publishing Group.

Clinically, HCC tumors display a marketable response to ICB therapy, which can significantly prolong the survival of HCC patients when combined with conventional treatments such as chemotherapy, radiotherapy, and targeted therapy. However, many patients lost their response to such a combination therapy due to the ITM and insufficient tumor immunogenicity.^[^ ^151^ ^]^ To improve ICB therapy in HCC, Xiao et al. focused on restoring p53 expression in HCC cells by targeted delivery of mRNA encoding the tumor suppressor gene p53.^[^ ^152^ ^]^ The p53 protein can transcriptionally regulate the expression of key cytokines (e.g., TNF‐α, IL‐12, and IL‐15), chemokines (e.g., CCL2, −20, and −28), and pathogen recognition receptors (e.g., TLRs) that regulate the interaction between tumor cells and immune cells to reverse the ITM (Figure 7A).^[^ ^153^ ^]^ They designed a lipid‐polymer hybrid NP for targeted delivery of mRNA: the interior is a core formed by biocompatible PLGA polymer and G0‐C14/mRNA complexes, and the surface is a lipid‐PEG layer. In addition, the PEG on NP surface was conjugated with CTCE protein, which can target and bind to the HCC‐specific protein CXCR4, achieving highly selective delivery to HCC cells both in vivo and in vitro. The platform with or without combination with anti‐PD1 achieved high expression of p53 in RIL‐175 cells, confirming the feasibility of combining p53 mRNA with ICB therapy (Figure 7B). When combined with ICB therapy, it effectively promoted tumor antigen‐specific adaptive immunity and inhibited the growth of HCC tumors, and significantly prolonged the survival of tumor‐bearing mice (Figure 7C,D). The study demonstrated that restoring p53 function based on an mRNA‐targeted delivery nano‐platform may provide an opportunity to reverse the ITM and improve the antitumor efficacy of ICB therapy.

Kong et al. also restored p53 expression by delivering p53‐mRNA in redox‐responsive NP and increased the sensitivity of p53‐null HCC and NSCLC cells to everolimus, an anti‐tumor small molecule chemotherapeutic drug.^[^ ^154^ ^]^ Moreover, to elicit a more substantial tumor‐killing effect against triple‐negative breast cancer (TNBC), Zhang et al. combined paclitaxel (PTX) with p53‐mRNA via PTX amino lipid (PAL) derived NPs.^[^ ^150a^ ^]^ These chemotherapy drug‐derived NPs displayed synergic cytotoxicity against TNBC cells and significantly inhibited tumor growth in vivo, showing the potential of this combinational therapy. Above advances suggested that the combination of p53 restoration and ICB therapy may be a revolutionary treatment for HCC and other p53‐deficient cancer.^[^ ^152^ ^]^

Similarly, Shi's group restored the expression of another tumor suppressor gene, the phosphatase and tensin homolog deleted on chromosome 10 (PTEN), by transporting PTEN‐mRNA to PTEN‐mutated melanoma cells via PLGA‐based NPs.^[^ ^155^ ^]^ In vivo results revealed that this platform reversed ITM by enhancing the expression of proinflammatory cytokines and CD8^+^ T cell infiltration in the tumor tissues. Combinational therapy with ICB agent elicits robust antitumor efficacy and long‐term immunological memory in the PTEN‐mutated melanoma mice model. Shi et al. reported a similar strategy to deliver PTEN‐mRNA with polymer‐lipid hybrid NPs.^[^ ^156^ ^]^ The constructed NPs were coated with PEG, which enhanced the serum stability and transfection efficiency of mRNA to prostate cancer cells. In prostate cancer‐bearing mice, PTEN was successfully expressed in cancer cells for inducing apoptosis to suppress tumor growth.

Chemokines like CCL2 and CCL5 are involved in the formation of ITM, which can induce TAM polarization toward the tumor‐promoting M2 phenotype. To this end, Wang et al. used MC3 LNP platform ^[^ ^123^ ^]^ to encapsulate mRNA encoding single‐domain antibody that binds and neutralizes CCL2 and CCL5 (BisCCL2/5i).^[^ ^124^ ^]^ After injection in an orthotopic HCC tumor model, the LNPs were mainly internalized by Hepa1‐6 tumor cells and achieved high expression of BisCCL2/5i, which significantly induced the polarization of TAMs toward the tumor‐inhibiting M1 phenotype and reverses immunosuppression in the TME. The BisCCL2/5i mRNA nano‐platform can also combine with PD‐1 inhibitor and prolongs survival time in mouse models of primary liver cancer, which broadens the combinational strategy of ICB therapy.

Delivering mRNA‐encoding cytotoxic proteins to tumor cells can directly kill tumor cells. For this purpose, it is essential to selectively provide mRNA to tumor cells only, ensuring that normal cells are not transfected and killed. Jain et al. provided a method to incorporate miRts (microRNA target sites) into the 3′UTR of modified mRNAs.^[^ ^157^ ^]^ miRts mediate a siRNA‐like cleavage mechanism to degrade mRNA in unintended recipient cells, solving the problem of expressing toxic proteins in normal cells. Specifically, they added miRts into the 3′UTR of mRNA encoding p53 up‐regulated modulator of apoptosis (PUMA, a key mediator of apoptosis) to control the protein expression in different cells. The results showed that PUMA was only expressed in HCC cells to induce apoptosis and normal cells were unaffected. This miR‐mRNA strategy opens up a novel approach of specifically expressing proteins in target cells with high precision, which holds great potential in mRNA‐based cancer immunotherapy.

Clinical studies have shown that high IL‐12 level benefits the recovery of HCC patients.^[^ ^158^ ^]^ However, there is currently a lack of platforms to selectively deliver IL‐12 to tumor tissues. Given this, Lai et al. designed LNPs to selectively deliver mRNA encoding IL‐12 to HCC cells, increasing the infiltration of activated immune cells (such as CD3^+^ CD4^+^ helper T cells) in tumors and effectively inhibiting the occurrence of HCC.^[^ ^127b^ ^]^ This study suggests that IL‐12‐LNP may be an effective immunotherapy against human HCC. Still, the impact of IL‐12 on other immune cells (such as macrophages and DCs) needed to be further studied. Yang et al. designed an ionizable lipid‐based LNP delivery system (composed of iBL0713, cholesterol, C16‐PEG, and mRNA) to encapsulate mRNA and form typically spherical NPs efficiently.^[^ ^159^ ^]^ This platform can selectively transport mRNA into hepatoma cells, reaching the highest expression of fluorescence peptidase or EPO about 6 h after administration. Amino‐ester lipid‐like material‐based LNP can also efficiently encapsulate mRNA and transfect HCC specifically in vivo. These studies provide an effective mRNA delivery strategy for treating liver‐related diseases such as anemia and HCC.^[^ ^58^ ^]^

**FIGURE 8:**
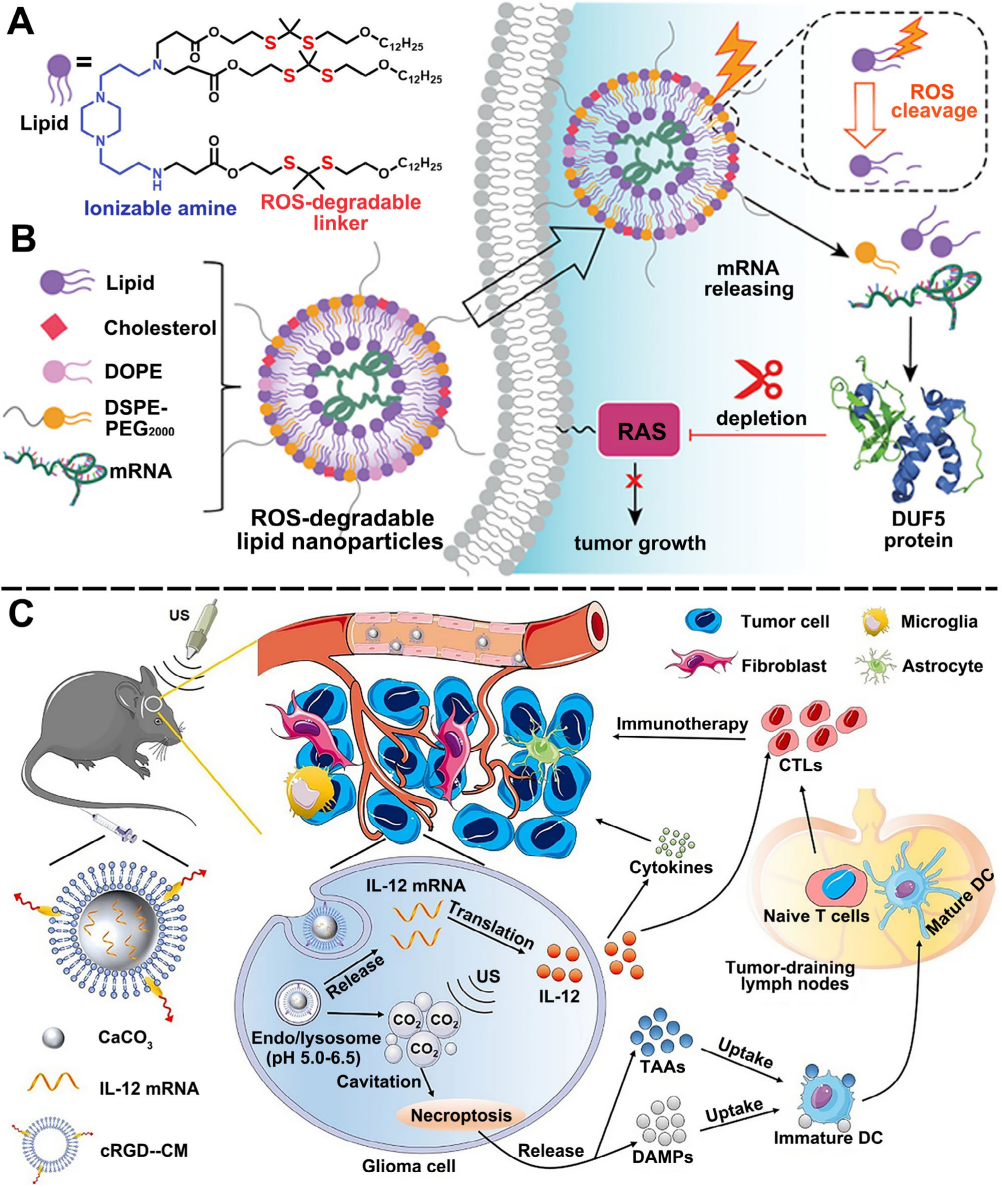
Schematic illustration of ROS‐degradable NPs for tumor cell target delivery of mRNA. A ROS‐degradable chemical structure of lipid showing ionizable amines and ROS‐responsible linkers. B Schematic illustration of the preparation of ROS‐degradable lipid NP for delivering mRNA‐encoding DUF5 to cleave RAS and inhibit tumor growth. Reproduced with permission.^[^ ^160^ ^]^ Copyright 2022, John Wiley and Sons. C Schematic illustration of the activity of IL‐12mRNA‐cRGD‐CM‐CaCO3 NPs in cavitation‐induced necroptosis and IL‐12‐activated cancer immunotherapy. Reproduced with permission. Copyright 2022,^[^ ^161^ ^]^ Springer Nature.

Apart from targeting HCC cells, many studies delivered mRNA to other tumor cells for cancer immunotherapy. For example, Ren et al. developed a vitamin E succinate‐modified polyethylenimine‐based self‐assembled polymeric micelle that forms complexes with mRNA via electrostatic interaction.^[^ ^106^ ^]^ Compared to other cell lines, the system selectively delivered mRNA to HeLa cervical tumor cells and elicited efficient protein expression to fight tumors. Similarly, Cai et al. reported a reactive oxygen species (ROS)‐degradable LNPs via screening a library of synthetic lipids containing a thioketal (TK) moiety and ionizable amines (whose protonation in acidic endosomes facilitates endosomal escape) to selectively deliver mRNA to Hela cells and achieved highly efficient protein expression (Figure 8A,B).^[^ ^160^ ^]^ ROS is a particular cancer hallmark and more prominent in tumor cells than normal cells, which was used to design a spatiotemporally controlled mRNA‐based platform in the study. As a result, the delivered mRNA encodes DUF5, a bacterial‐derived RAS protease,^[^ ^162^ ^]^ which cleaves the conserved domain of RAS and significantly inhibits tumor growth.

Cytokines like IL‐12 can also be applied for GBM and melanoma therapy. To precisely deliver IL‐12‐mRNA into glioma cells for GBM treatment, Zhao et al. coated mRNA‐CaCO_3_ NPs with cell membrane (CM) of GL261 cells, which plays the homotypic target effect.^[^ ^161^ ^]^ Moreover, the CM was previously labeled with Cyclic Arg‐Gly‐Asp (cRGD), a peptide that could bind to integrin overexpressed in GBM neo‐vasculature, for passing through the blood‐brain barrier. As expected, CaCO_3_ was decomposed at acidic pH conditions in tumor cells to produce IL‐12‐mRNA and CO_2_, which could induce a cavitation effect for necroptosis under ultrasound treatment. Meanwhile, the released mRNA‐translated IL‐12, together with the damage‐associated molecular patterns derived from necroptosis, could potently activate T cells for efficient cancer immunotherapy (Figure 8C).

IL‐12‐mRNA could be used to treat melanoma as well. Liu et al. synthesized a series of ionizable lipids (DAL1‐DAL7) containing di‐amino groups with various head groups. They found that LNP containing DAL‐4 could directly deliver mRNA encoding immune‐stimulating IL‐12 to tumor cells.^[^ ^50^ ^]^ Their results showed that after administration, cytokines such as IL‐12, IL‐27, and GM‐CSF were successfully expressed in B16F10 melanoma tumor cells. These cytokines subsequently induced intense infiltration of immune effector cells like NK and CD8^+^ T cells and significantly inhibited tumor growth in B16F10 melanoma tumor‐bearing mice. The LNP‐based delivery of cytokine‐mRNA to tumor cells provides a novel strategy for cancer immunotherapy.

**FIGURE 9:**
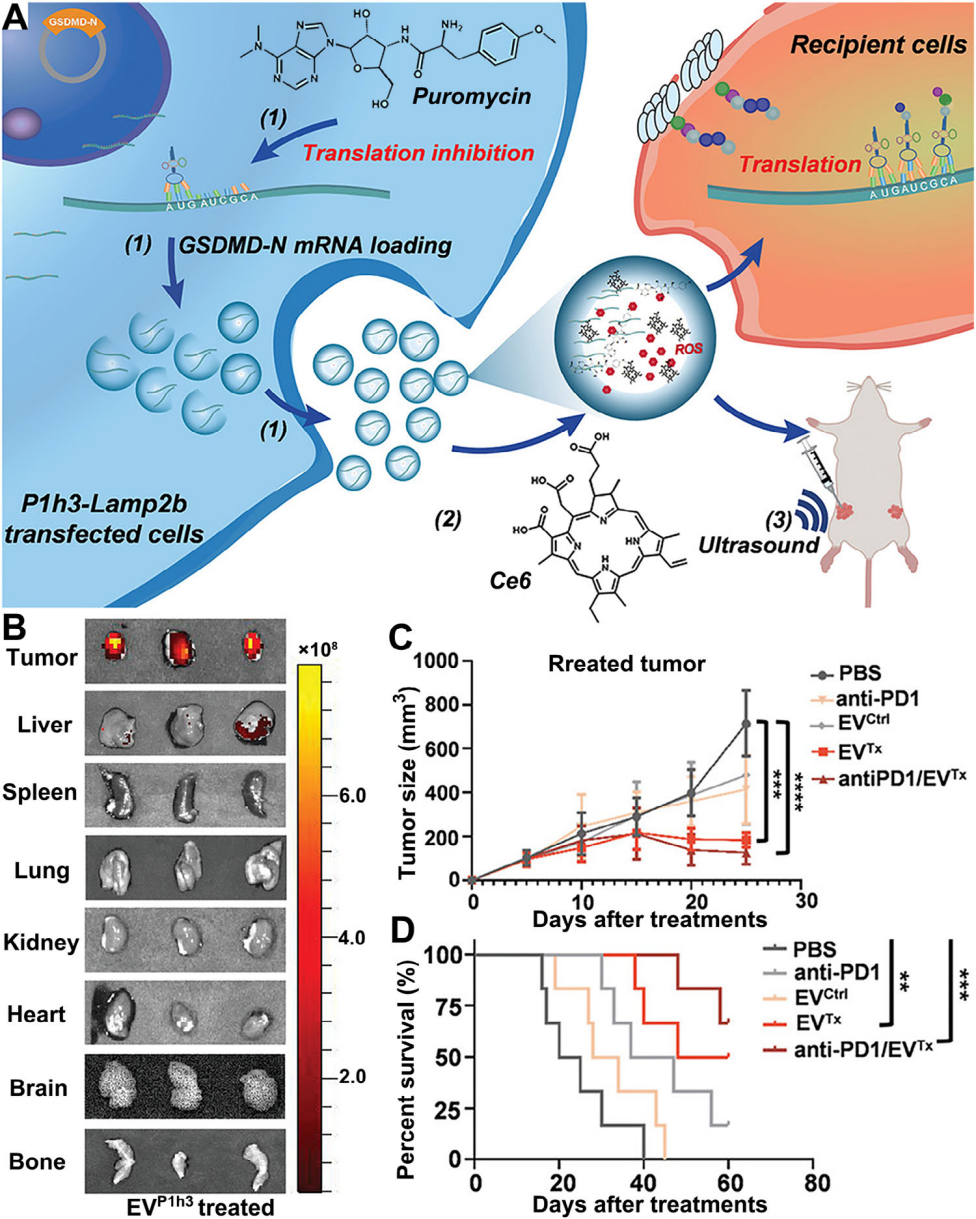
Schematic illustration of engineered EVs for tumor cell‐targeted GSDMD‐N mRNA delivery to treat cancer. A GSDMD‐N mRNA was encapsulated in EVs by vesicle donor cells and Ce6 was incorporated into the vesicles for sonodynamic treatment. B Ex vivo images of DiR‐labeled EV distribution at 4 h after intratumoral injection. C,D Tumor size and survival curves of 4T1 breast tumor‐bearing mice after being treated with different groups. Reproduced with permission.^[^ ^164^ ^]^ Copyright 2023, John Wiley and Sons.

Biomembrane‐derived delivery materials were also employed to specifically transport mRNA to tumor cells. Zhang et al. designed a platform of gold NPs (AuNPs) with leukemia cell‐membrane vesicle modification, which can specifically target leukemia cells after systemic administration, providing an approach to treating leukemia via selectively delivering mRNA to leukemia cells.^[^ ^163^ ^]^ Xing et al. innovatively designed a multifunctional EVs‐based delivery system through engineering approaches.^[^ ^164^ ^]^ Specifically, the mRNA of GSDMD‐N, a key executing molecule of pyroptosis, was transcripted in donor cells using a synthetic plasmid as template and subsequently encapsulated into EV with puromycin repressing the premature translation of mRNA. Chlorin e6 (Ce6, a photosensitizer) was incorporated into the membrane of EV to initiate the translation of GSDMD‐N‐mRNA in tumor cells by inactivating puromycin through sonodynamic treatment. Ce6 can also induce immunogenic cell death, which elicits potent tumor immunotherapy along with GSDMD‐N‐induced pyroptosis (Figure 9A). To achieve the targeted delivery of mRNA, they armed the EV with HER2 antibody (P1h3), which can facilitate the tropism to HER2^+^ breast cancer cells. As a result, in the HER2^+^ breast tumor cell‐bearing mouse models, the engineered mRNA‐EV was retained in tumor tissues specifically (Figure 9B) and significantly inhibited tumor growth and prolonged survival time after being combined with anti‐PD1 therapy (Figure 9C,D). The study provided a promising strategy for combining mRNA with pyroptosis for robust cancer immunotherapy.

Tumor cell‐targeted delivery of mRNA can also be achieved by directly injecting naked mRNA into the tumor sites. Hoecke et al. described that intra‐tumoral injection of mRNA encoding necroptosis executioner mixed lineage kinase domain‐like (MLKL) protein could be a promising antitumor therapy.^[^ ^165^ ^]^ They used electroporation after administration to facilitate the uptake of mRNA by CT‐26 colorectal tumor cells. The expressed MLKL evoked tumor cell death and attracted Batf3‐dependent DCs, which can recognize tumor‐specific antigens and induce CD4^+^ and CD8^+^ T cell activation.

### 4.3 Dendritic cell‐targeted mRNA delivery

Dendritic cells (DCs) are the principal APCs that capture, process, and present antigens to T cells in the periphery, which is crucial for inducing an adaptive antigen‐specific immune response to fight heterogeneous microbes or tumor cells.^[^ ^166^ ^]^ DC, often called “natural adjuvant,” has become the natural medium of antigen transmission, which has two functions of immune response and immune tolerance to play an essential role in maintaining immune balance. DCs recognize PAMPs through PRRs like TLR‐7 and −8 on the cell surface, and process and present antigens by MHC‐I or ‐II for activating CD8^+^ T and CD4^+^ T cells respectively.^[^ ^167^ ^]^

Considering the superiorities of mRNA and the function of DCs, it is reasonable to deliver mRNA encoding TAAs to DC to trigger an antigen‐specific immune response to treat cancer. For example, Meulien's group first delivered mRNA encoding antigen to DC in vivo through a liposome‐based preparation and opened the prelude of DC targeted delivery of mRNA.^[^ ^168^ ^]^ Rein et al. also used a liposomal formulation composed of DOTAP and cholesterol, termed Galsomes, to selectively deliver OVA‐encoding mRNA to DC and activate substantial antigen‐specific cytotoxic T cells.^[^ ^45^ ^]^ Nguyen et al. demonstrated that NPs based on mesoporous silica materials (MSNs) could be taken up by DC after subcutaneous injection.^[^ ^1e^ ^]^ Their results showed that MSNs could be a rational delivery system of mRNA to DCs for antigen‐specific immune response to eradicate cancer in vivo. Tateshita et al. reported an mRNA‐based DC‐vaccine composed of an ionizable lipid‐like material with vitamin E‐scaffolds and an α‐helical cationic peptide “KALA.” The vaccine achieved high production of antigens and proinflammatory cytokines in murine bone marrow‐derived DCs (BMDCs), suggesting a robust ex vivo DC‐based RNA vaccine platform.^[^ ^115^ ^]^

**FIGURE 10:**
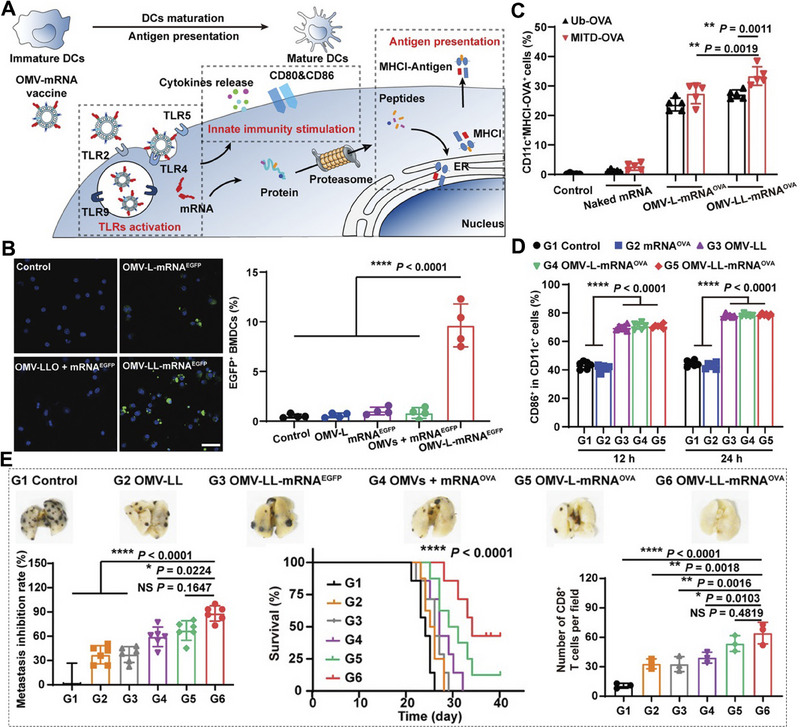
Bacteria‐derived outer membrane vesicles (OMV) for mRNA carriage to DCs to induce a potent and long‐term immune response. A Schematic illustration of the OMV‐based mRNA vaccine triggering TLR activation, innate immunity stimulation, and antigen presentation. B Expression of EGFP in DCs incubated with OMV‐LL‐mRNAEGFP and other formulations. C,D DC maturation and the expression of the MHCI‐OVA complex in DCs induced by various formulations. E Representative images of lungs, metastasis inhibition rate, and survival curves by OMV‐LL‐mRNAOVA and other groups in metastatic B16‐OVA tumor models. Reproduced with permission.^[^ ^169^ ^]^ Copyright 2022, John Wiley and Sons.

To improve the DC‐targeting efficacy of the mRNA delivery systems, Li et al. employed bacterial‐derived outer membrane vesicles (OMVs) as an mRNA delivery platform, and used L7Ae (an RNA‐binding protein capable of adsorbing the C/D box of modified mRNA) and a lysosomal escape protein, Listeria lysin O (OMV‐LL) to modify the surface of OMV (Figure 10A).^[^ ^169^ ^]^ Injected in metastatic B16‐OVA tumor‐bearing mice models, OMV‐LL selectively delivered mRNA to DCs with high efficiency. Then, the mRNA entered the cytoplasm of DC through Listeria hemolysin O‐mediated endosomal escape, where it expressed antigens efficiently (Figure 10B,C). Their results showed efficient DC maturation and subsequent tumor infiltration of CD8^+^ T cells, which induced a strong and durable antitumor immunological reaction. Moreover, OMV‐LL‐mRNA significantly inhibited lung metastasis after administration, which may be related to the innate immunity elicited by the PAMPs of bacterial components of OMV (Figure 10D,E).

Unlike the LNP delivery platform, the OMV‐based delivery system they constructed efficiently delivered mRNA tumor antigens to DCs for successful antigen processing and presentation. Moreover, the engineered OMVs possess intrinsic immunogenicity and play the role of an adjuvant that can effectively activate multiple TLRs and the innate immune system. In addition, they provided a new loading strategy for mRNA based on the surface adsorption of L7Ae protein and the C/D box of modified mRNA. Their study is expected to accelerate the development of mRNA‐based cancer immunotherapy.

NPs have been extensively applied in the DC‐targeted delivery of mRNA. In this paradigm, Krienke et al. systematically delivered mRNA‐encoding disease‐related autoantigens to splenic CD11c^+^ APCs via an NP formulation.^[^ ^48c^ ^]^ The NP achieved antigen presentation on DCs and the expansion of antigen‐specific CD4^+^ T cells in a noninflammatory context for autoimmune disease treatment.

LNPs can also be modified by targeting modules like antibodies to improve their in vivo distribution selectivity.^[^ ^170^ ^]^ Katakowski et al. coated LNPs with a single‐chain antibody specific to murine DEC205, a marker of DCs95.^[^ ^171^ ^]^ They found that the constructed anti‐DEC205 scFv‐modified LNPs could specifically target DEC205^+^ DCs. However, modifying LNPs with functional molecules implies increasing the cost and time of production, and the possibility of losing target ability in the complex biological environment.^[^ ^172^ ^]^ Therefore, the strategy of modifying LNPs should be carefully adopted in mRNA delivery in vivo. Besides, reports showed a clear correlation between the particle sizes of LNPs and their DC targeting and cellular uptake efficiency,^[^ ^173^ ^]^ which necessitates researchers to consider the size effect of mRNA‐based LNPs.

To explore the feasibility of mRNA‐based immunization bypassing the injection administration in tumor treatment, Wang et al. used ethosomes to construct a transcutaneous immunization system to deliver mRNA‐encoding tyrosinase‐related protein 2 (TRP2, a model of TAAs) and siRNA against PD‐L1.^[^ ^174^ ^]^ The system can efficiently transfect DCs, express protein TRP2, and significantly inhibit melanoma tumor growth by improving the infiltration of CD4^+^ and CD8^+^ T cells.

All the examples discussed above are focused on delivering TAA‐mRNA to DCs to elicit DC maturation and subsequent antigen presentation. The breakthrough in cancer treatment may lie in keeping pace with cancer neoantigen discovery when constructing DC‐targeted mRNA‐TAA platforms.^[^ ^175^ ^]^ Furthermore, uprising proteins that are essential for the lifespan and maturation of DC (e.g., Akt1) with mRNA may represent a promising strategy for enhancing cancer immunotherapy.^[^ ^176^ ^]^ DC‐targeted delivery of Akt1‐mRNA can level up Akt1 in DC, thus eliciting long‐term memory responses to fight cancer.

### 4.4 T cell‐targeted mRNA delivery

ICB therapy holds promise for many patients with refractory cancers by blocking T cell co‐suppressive pathways, such as PD‐1/PD‐L1 and CTLA‐4.^[^ ^177^ ^]^ However, the clinical efficacy of ICB therapy alone is not enough to eradicate tumor cells. Researchers have attempted to activate T cells by stimulating the costimulatory receptors of T cells (such as CD137 and OX40) to facilitate ICB therapy. Recently, antibodies such as anti‐OX40 and anti‐CTLA‐4 antibodies, targeting co‐stimulatory receptors of T cells have been well‐developed, and have achieved specific curative effects.^[^ ^178^ ^]^ However, the expression level of costimulatory receptors on T cells is insufficient to produce a satisfying curative outcome.

**FIGURE 11:**
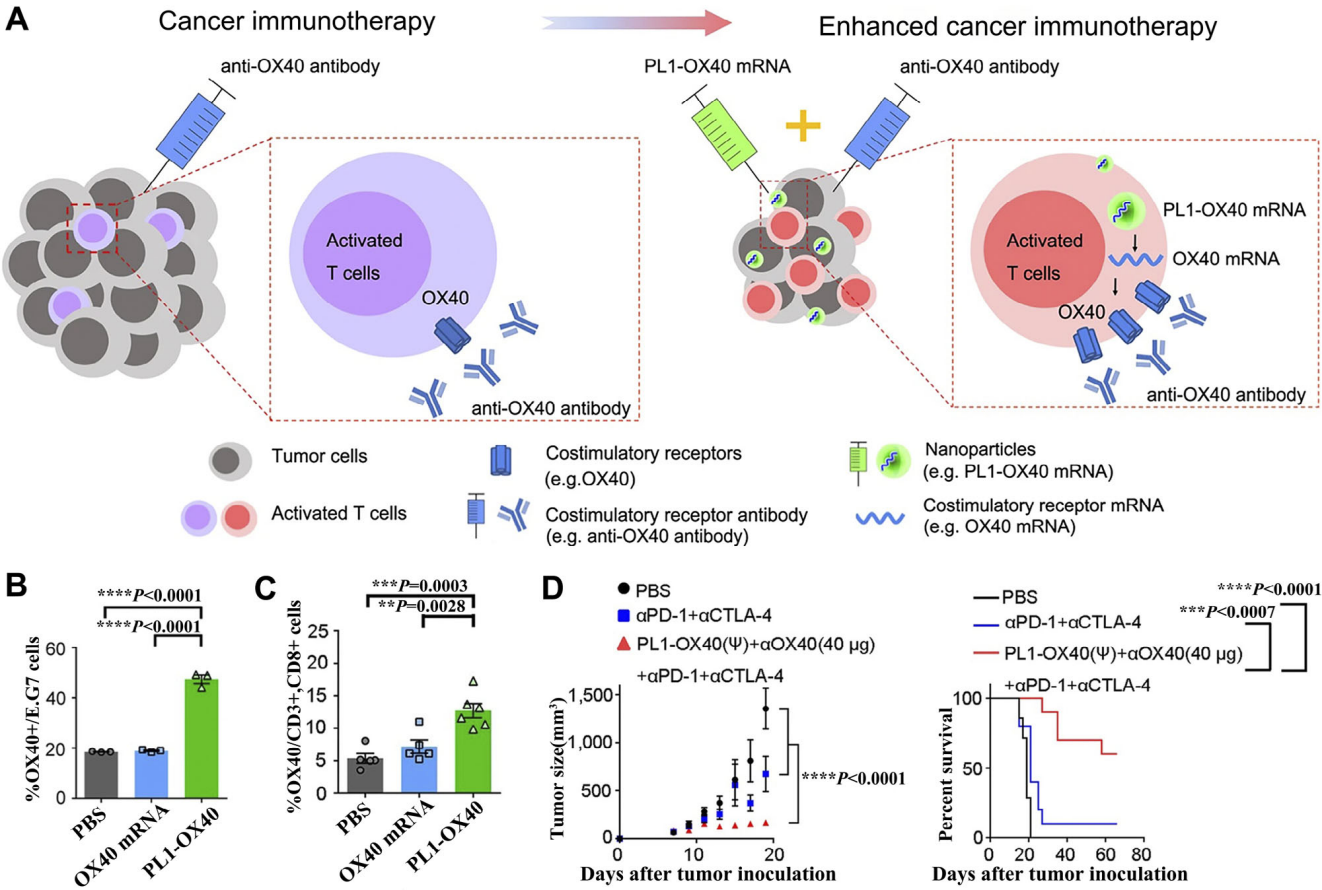
Biomimetic phospholipid NPs transport mRNA to T cells for cancer immunotherapy. A Schematic representation of biomimetic NPs delivering OX40 mRNAs for enhancing T cell‐mediated cancer immunotherapy. B PL1‐OX40 induced OX40 expression in E.G7 cells (a T‐lymphocyte cell line). C OX40 expression on the CD8^+^ T cells in the A20 B cell lymphoma model after treatment with PL1‐OX40. D Tumor volumes and overall survival curves of A20 tumor‐bearing mice after treatment with PL1‐OX40+anti‐OX40 antibody+anti‐PD‐1+anti‐CTLA‐4. Reproduced with permission.^[^ ^117^ ^]^ Copyright 2021, Nature Publishing Group.

From the perspective of mRNA‐based immunotherapy, Li et al. designed a biomimetic phospholipid nanoparticle (PL1) with T‐cell target ability to deliver mRNA encoding co‐stimulatory receptors (CD137 or OX40).^[^ ^117^ ^]^ This strategy significantly increased the expression level of costimulatory receptors in tumor‐infiltrating T cells (Figure 11A–C). Compared with agonists of co‐stimulatory receptors or ICB inhibitors alone, the combination therapy of co‐stimulatory receptor mRNA with the corresponding co‐stimulatory receptor agonists (anti‐OX40 and anti‐CTLA‐4 antibodies) and ICB inhibitors (anti‐PD‐1 + anti‐CTLA‐4 antibodies) significantly improved the antitumor immune response and prolonged the survival time in B16F10 tumor‐bearing mice (Figure 11D). Moreover, co‐stimulatory receptors are also expressed on DCs. In the study, PL1‐OX40 NPs also increased OC40 expression on the surface of DCs, and subsequent activation of DCs to stimulate T cell activation.

Modifying cholesterol with hydroxyl groups can alter the endocytic recycling mechanisms of LNP, thus enhancing the targeted delivery of encapsulated mRNA.^[^ ^179^ ^]^ Patel et al. substituted 50% cholesterol of LNP by hydroxycholesterols and found that the T cell targeted delivery efficiency of LNP‐mRNA increased about twofold, suggesting that hydroxycholesterol substitution for cholesterol in LNPs is a promising method of mRNA‐based T cell therapeutics.^[^ ^88^ ^]^ Billingsley et al. optimized the excipient molar ratios of LNP via the orthogonal design of experiments methodology.^[^ ^89^ ^]^ They obtained LNP (termed B10) that could deliver CD19‐specific CAR mRNA to T cells with lower cytotoxicity than electroporation and a threefold increase in delivery efficiency compared to standard LNP formulation. When cocultured with Nalm‐6 ALL tumor cells, B10 LNPs‐treated CAR‐T cells showed comparable tumor cell killing ability with electroporation, the clinical standard for CAR mRNA delivery. These results suggested that the B10 LNP platform is a promising vehicle for T‐cell engineering applications. However, the mechanism of how excipient compositions enhance delivery still needs exploration.

Considering the complex internal environment, the delivery of mRNA into T cells in vivo may not be entirely consistent with in vitro observations. Zhao et al. synthesized a library of imidazole‐containing lipidoids, which showed potent mRNA transfection efficiency in T cells in vitro.^[^ ^180^ ^]^ To investigate whether the optimized LNP could also work in vivo, they intravenously delivered LNPs containing Cre recombinase mRNA into Ai14 mice. Ai14 mice can express red tdTomato once Cre recombinase excises the STOP codon between the loxP sites. Strong tdTomato signal was observed in spleen T cells with an 8.2% gene recombination rate in mice, suggesting the imidazole‐based lipidoids could be a proper mRNA‐based platform in T cell engineering.

**FIGURE 12:**
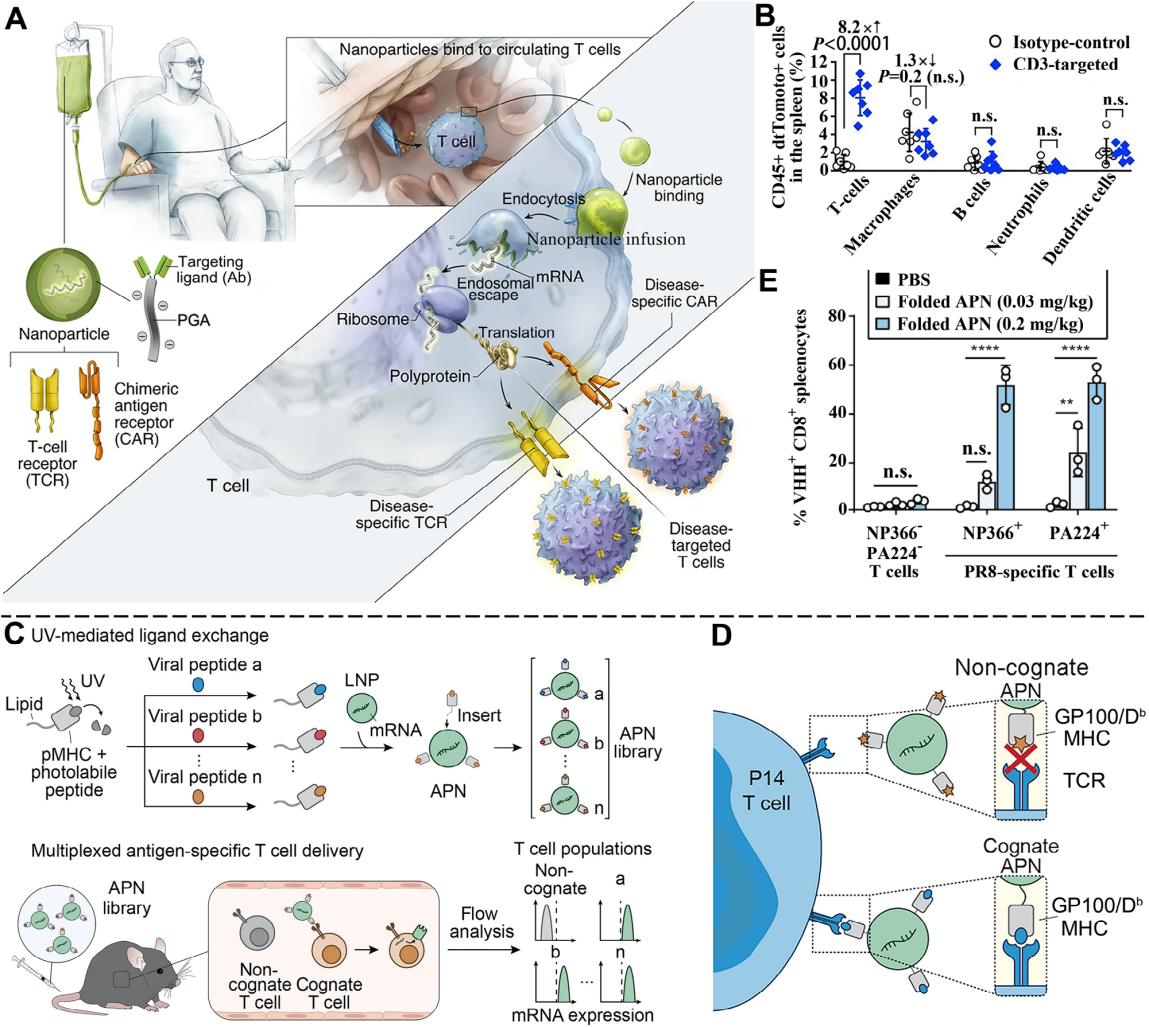
Schematic representation of T cell‐targeted delivery of mRNA via Anti‐CD8 antibody coated PBAE‐based NPs and MHC‐I antigen‐presenting NPs. A PGA‐antibody coated PBAE‐based NPs deliver mRNA encoding TCR or CAR specifically to circulating T cells to fight tumor cells. B PGA‐antibody‐coated NPs deliver mRNA to T cells preferentially. Reproduced with permission.^[^ ^181^ ^]^ Copyright 2020, Nature Publishing Group. C UV‐mediated exchange of photolabile peptides with viral peptides on MHC endows NPs with the ability to target cognate T cells. D Schematic illustration of MHC‐antigen on LNP in mediating targeted delivery of mRNA to cognate T cell. E MHC‐antigen‐modified LNP delivers mRNA encoding VHH preferentially into cognate T cells. Reproduced with permission.^[^ ^184^ ^]^ Copyright 2022, American Association for the Advancement of Science.

In addition to adjusting the compositions of LNPs, modifying LNPs with antibodies or antigen phenotypes that bind to specific receptors on T cells can also achieve the aim of T cell‐target delivery of mRNA with higher targeting precision. Parayath et al. self‐assembled anionic mRNA with cationic PBAE to form NP and then coated it with anti‐CD8 antibodies via polyglutamic acid (PGA).^[^ ^181^ ^]^ The antibody on the surface of NPs can actively target CD8 on circulating T cells and the delivered mRNA encoding CAR or TCR transfect T cells through endocytosis (Figure 12A,B). The translated tumor‐specific CAR or virus‐specific TCR can locate onto the membrane and reprogram circulating T cells to eliminate tumors or external infections.^[^ ^182^ ^]^ The report provided an approach for conveniently programming T cells in vivo, which can be an ideal alternative for manufacturing engineered T cells ex vivo.^[^ ^183^ ^]^

To achieve T cell‐targeted mRNA delivery with higher specificity, Su et al. designed a UV‐mediated peptide exchange on lipid‐modified MHC‐I, which can be inserted in mRNA‐loaded NPs via lipid‐mediated hydrophobic interactions (Figure 12C).^[^ ^184^ ^]^ After intravenous injection, the constructed MHC‐I antigen‐presenting NPs can specifically target cognate CD8^+^ T cells via the recognition of antigen phenotype with TCR (Figure 12D). Using mRNA encoding a single variable domain on a heavy chain (VHH) antibody, they demonstrated that the antigen‐presenting NPs could elicit VHH expression in cognate CD8^+^ T cells rather than the noncognate counterparts (Figure 10E). The UV‐mediated peptide exchange significantly promotes the potential of preparing different antigen‐presenting NPs to express targeted proteins in specific T cells via mRNA.

Naked mRNA can also directly transfect T cells in vitro to obtain cytotoxic CD8^+^ T cells (CTL), which could be injected back into the body to fight tumor cells.^[^ ^185^ ^]^ In this regard, Wen et al. used single‐cell RNA‐sequencing (scRNA‐seq) technology to compare the transcription map of CD8^+^ T cells from the peripheral blood of complete responders and non‐responders to anti‐PD‐1 therapy and found that NKG7 (cytolytic granule‐associated molecule natural killer cell granule protein‐7) was down‐regulated in non‐responders.^[^ ^186^ ^]^ Mechanistic studies revealed that NKG7 promotes the antitumor effect of T cells by altering cytolytic granule number, trafficking, and calcium release. To restore the function of NKG7, they transfected T cells with NKG7 mRNA, improved the cytotoxic ability of CTL isolated from non‐responders, and increased their response to PD‐1/PD‐L1 therapy. To inhibit the peritoneal dissemination of melanoma and pancreatic cancer, Trani et al. intraperitoneally administrated T cells that were pre‐electroporated with IL‐12 mRNA.^[^ ^187^ ^]^ Their results showed that T cells homed to the omentum effectively and suppressed the development of tumors spread in the peritoneal cavity.

### 4.5 B cell‐, macrophage‐, and other cell‐targeted mRNA delivery

B lymphocytes (termed B cells) play an irreplaceable role in regulating body immunity. They can produce and secrete antibodies and cytokines, and present antigens through MHC I and MHC II to promote T cell activation. Therefore, regulating the function of B cells in vivo has great potential for disease prevention and treatment.

**FIGURE 13:**
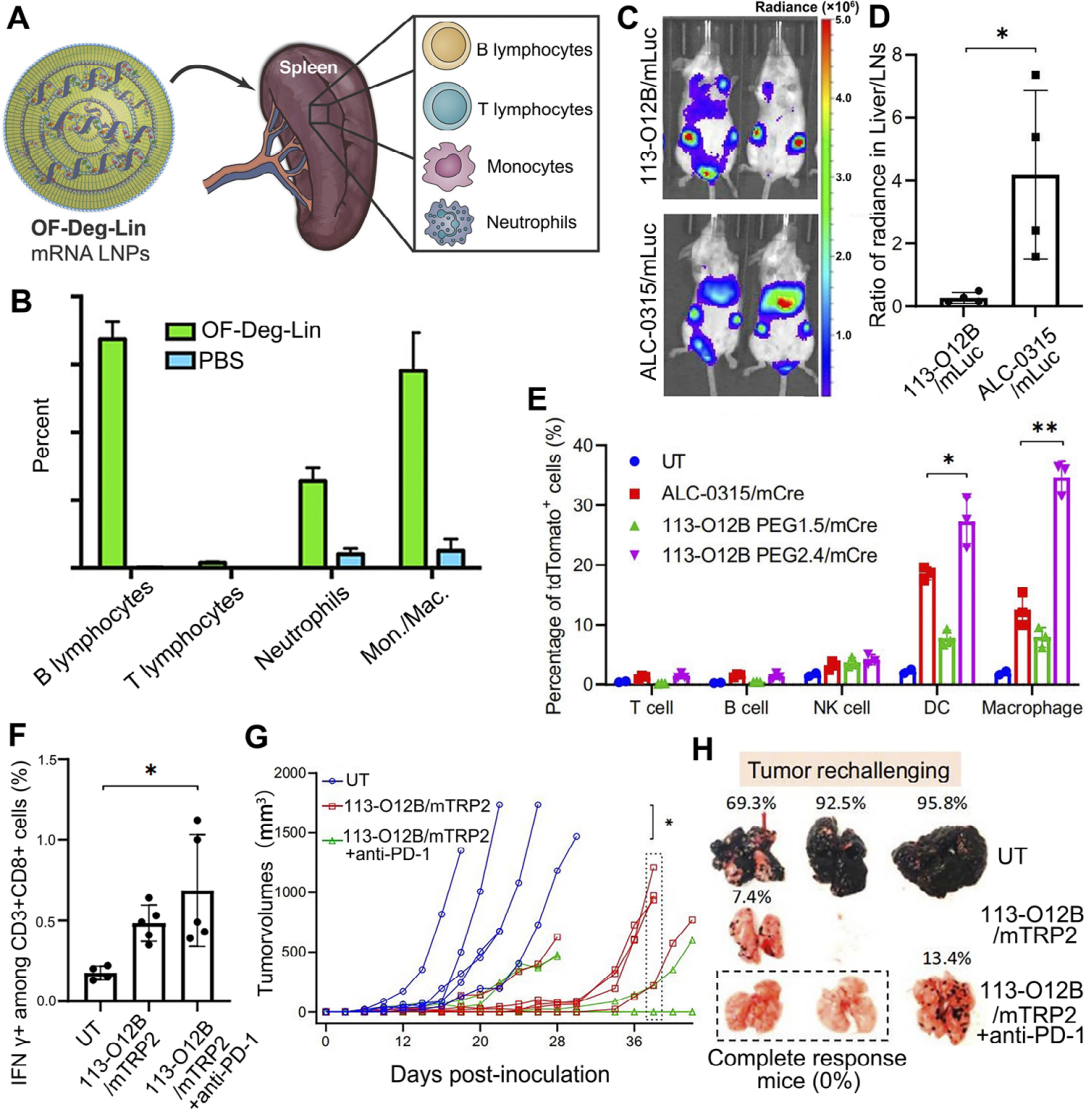
Schematic illustration of transporting mRNA to B lymphocytes to induce potent immune‐associated molecule production and to macrophages to elicit robust CD8+ T cell response. A OF‐Deg‐Lin LNPs deliver mRNA to immune cells including B lymphocytes in spleen. B The percentage of cells labeled with OF‐Deg‐Lin Cy5 mRNA LNPs. Reproduced with permission.^[^ ^188^ ^]^ Copyright 2017, John Wiley and Sons. C,D 113‐O12B LNP increased the expression of luciferase mRNA (mLuc) in LNs than ALC‐0315. E 113‐O12B LNP delivers mRNA preferentially to APCs including macrophages and DCs. F 113‐O12B LNP encapsulating TRP2_180‐188_ mRNA elevated the amount of IFN‐γ^+^ cells within CD8^+^ T cells in PBMCs. G,H The combinatory strategy of 113‐O12B/mTRP2 with anti‐PD‐1 significantly inhibits tumor growth and metastasis in B16F10 tumor‐bearing mice. Reproduced with permission.^[^ ^189^ ^]^ Copyright 2022, National Academy of Sciences.

Considering the significant advantages of mRNA in protein expression, researchers envisioned increasing the expression level of functional proteins in B cells by selectively delivering mRNAs into B cells. Fenton and his colleagues developed a synthetic ionizable lipid‐based LNP system capable of encapsulating aimed mRNAs and delivering them to the spleen, efficiently transfecting B cells, and inducing efficient protein expression in the spleen (Figure 13A,B).^[^ ^188^ ^]^ The ionizable lipid OF‐Deg‐Lin synthesized by a three‐step reaction contains electrophilic ester bonds that are more easily degraded in the spleen, which partly explains why LNPs distributed to other organs exhibited no protein expression. However, the structure–activity relationship of OF‐Deg‐Lin still needs further exploration, which may provide meaningful help for B cell‐targeted drug delivery.

After long‐term interaction with TME, TAMs generally develop into two phenotypes: the M1 type which is considered beneficial and acts as a scavenger of pathogens, and the M2 type which has the negative effect of suppressing immunity and promoting tumor development.^[^ ^65^ ^]^ Unfortunately, TAMs in most human tumors are mostly M2 type, which motivates tumor development, metastasis, and chemotherapeutic resistance. Researchers envisioned reprogramming M2‐type macrophages into M1‐type macrophages would inhibit tumor development.

Recently, studies have shown that substances like IL‐12, IFN‐γ, TLR agonists, and CD40 agonists can induce TAM repolarization to M1‐type.^[^ ^190^ ^]^ However, these agonists have inevitable dose‐dependent side effects after systemic administration. To address this issue, Zhang et al. designed NPs that selectively delivered mRNAs encoding macrophage polarization factors to M2 macrophages and reduced the systemic toxicity caused by off‐target effects.^[^ ^191^ ^]^ Specifically, they synthesized the NPs with cationic PBAE polymers and Di‐mannose. PBAE binds to negatively charged mRNAs through electrostatic interactions, while Di‐mannose is grafted onto the surface of NPs through PGA to target MRC1 (a membrane protein specifically expressed on M2 macrophages). The results showed that their synthesized NPs successfully delivered mRNAs encoding interferon regulatory factor 5 (IRF5) and IKKβ (a kinase that phosphorylates and activates IRF537) to M2‐type TAMs, eliciting highly efficient targeted protein expression. The obtained factors reprogrammed most M2‐type macrophages into M1‐type to fight tumor cells, significantly improving survival time of ovarian cancer‐bearing mice.

Macrophages can also act as an APC for antigen presentation, implying mRNA encoding TAAs can be delivered to macrophages for enhanced cancer immunotherapy. Chen et al. synthesized a lipid library and selected the top‐performing lipid, 113‐O12B, for mRNA in vivo LNs‐targeted delivery.^[^ ^189^ ^]^ The 113‐O12B possesses an ester bond linker and a short tail. Compared with LNP formulated with ALC‐0315, an approved standard lipid for COVID‐19 vaccine delivery, 113‐O12B transported luciferase mRNA (mLuc) preferentially to LN rather than the liver, demonstrating its target ability to LN (Figure 13C,D). At the cellular level, 113‐O12B delivered Cre mRNA to APCs specifically, reaching tdTomato expression in ∼34% of macrophages (Figure 13E). To evaluate the antitumor ability of this platform, they used 113‐O12B LNP to transport mRNA encoding TRP2, which exhibited accumulation in LNs and antigen expression in macrophages, much higher than in other cell types. This platform induced robust CD8^+^ T cell response (Figure 13F) and hindered the development and metastasis of tumors in B16F10 melanoma mice model (Figure 13G,H). The 113‐O12B‐based LN‐ and APC target delivery vehicle can improve the antigen presentation on MHC‐I molecules and subsequent immune response activation, representing a novel strategy for cancer immunotherapy. Moreover, equipping LNPs with vitamin‐derived lipids can endow mRNA delivery with targeting ability for macrophages. Hou et al. synthesized a series of vitamin‐derived lipid NPs to precisely deliver mRNA encoding antimicrobial peptide IB367 (AMP) and cathepsin B (CatB) to macrophages, eliciting a substantial protecting effect against bacteria‐induced sepsis via reversing the immunosuppressive microenvironment of tumors.^[^ ^192^ ^]^

Apart from those cell types discussed above, many researchers delivered mRNA to other cells for specific protein expression to facilitate cancer therapy. Ni et al. tested the cell tropism of mRNA‐loaded LNP containing piperazine ionizable lipids (Pi‐Lipids) in 14 cell types in vivo.^[^ ^193^ ^]^ They found that LNP named Pi‐A10 can deliver mRNA into the liver and splenic immune cells preferentially. Similarly, Gan et al. quantified the mRNA‐delivering efficiency of 109 LNPs and found that the addition of conformationally constrained phospholipids can direct LNP to liver immune cells preferentially rather than hepatocytes.^[^ ^194^ ^]^ The study suggests that constrained phospholipids in LNP composition can be favorable to targeted mRNA delivery.

All the above examples are focused on strengthening the immune response to elicit robust antitumor efficacy. However, there are situations in which temporal and spatial immunosuppressive environments need to be obtained without impairing the integral immune system, especially when cancer patients get inflammation in body. This spatial immune manipulating strategy urgently needs targeted delivery of drugs like mRNA into the desired cell types. Inflammatory bowel disease (IBD) is a phlegmonosis associated with a dysregulated immune environment. To treat IBD without disturbing overall immune system, Veiga et al. selected interleukin 10 (IL10) as the anti‐inflammatory molecule and delivered mRNA encoding IL10 preferentially to Ly6c^+^ inflammatory leukocytes via anti‐Ly6c mAbs‐modified LNP formulations.^[^ ^195^ ^]^ They observed that IL10 was selectively translated in leukocytes of the colitis mice model, along with lower pro‐inflammatory cytokines like TNFα and IL6. The study demonstrated the potential to control the spatial immune environment in vivo, which holds great potential in treating inflammation of cancer patients.

## 5 IMAGING MONITORING OF MRNA‐BASED CANCER IMMUNOTHERAPY

**FIGURE 14:**
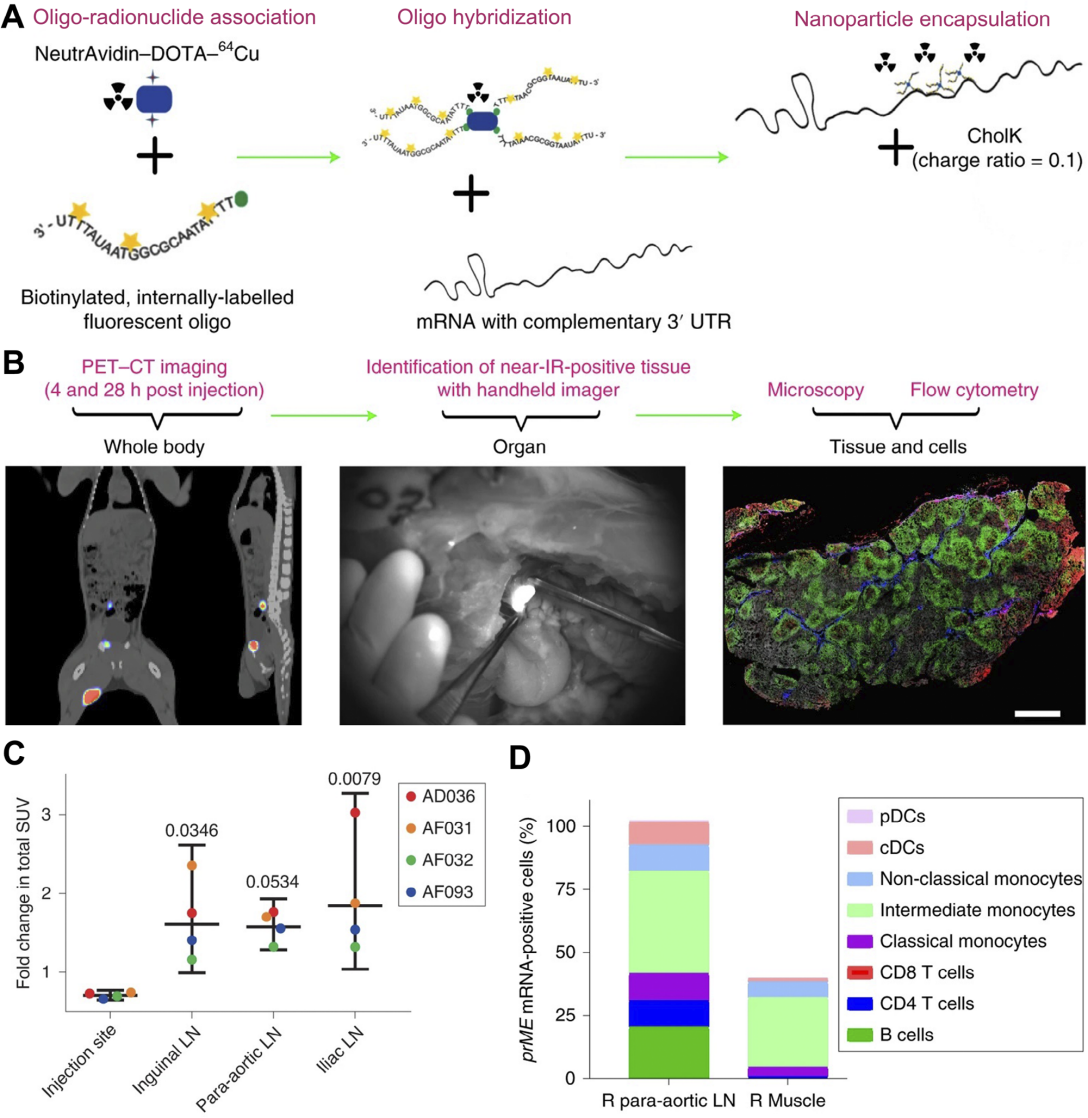
Whole‐body PET‐CT imaging and fluorescent labeling method of mRNA delivery in vivo. A Labeling mRNA with dual radionuclide‐near‐infrared probe. B Whole‐body PET‐CT imaging, mRNA‐positive tissues near‐infrared identification, and protein expression analysis after administration. C Fold change in total SUVs over 28 h in different sites of 4 cynomolgus macaques (AD036, AF031, AF032, AF093). D DCs and B cells accounted for the predominant labelled‐mRNA‐positive cell types. Reproduced with permission.^[^ ^196^ ^]^ Copyright 2019, Nature Publishing Group.

The spatiotemporal tracking of mRNA distribution after in vivo administration is crucial for evaluating the efficacy of candidate mRNA delivery systems, thus helping to accelerate the clinical translation of mRNA drugs. In addition, imaging of mRNA‐mediated protein expression in vivo will assist to evaluate the off‐target effects of delivery systems, which is of great significance for accelerating the development of platforms with targeting capabilities. However, most current experiments assessing the efficacy of mRNA drugs are based on host immune responses several days after administration, which is not timely enough. Also, optical imaging methods in large mammals, such as luminescence and fluorescence, are limited by the effects of light scattering. Therefore, there is an urgent need to develop non‐invasive methods for the timely monitoring of mRNA vaccine response and efficacy. Lindsay et al. directly labeled mRNA with orthogonal dual probes (^64^Cu and DyLight 680) for the in vivo imaging of mRNA.^[^ ^196^ ^]^ Specifically, mRNA was firstly labeled with fluorophore DyLight 680, then the 3′UTR of mRNA was labeled with tetravalent NeutrAvidin‐oligonucleotide complexes, and DOTA (a divalent cation chelator)−64Cu (the radionuclide PET reporter) complex was coupled to NeutrAvidin protein (Figure 14A). Upon injection into cynomolgus macaques, positron emission tomography‐computed tomography (PET‐CT) imaging and near‐infrared fluorescence imaging were employed to monitor mRNA distribution in vivo with a high spatiotemporal resolution (Figure 14B). The quantitative analysis of mRNA standard uptake value (SUV) dynamically monitored the flow of mRNA from the injection site to the draining LNs (Figure 14C). Besides, flow cytometry analysis showed that DCs and B cells are the predominant labelled‐mRNA positive cell types in para‐aortic LNs (Figure 14D). Excitingly, their labeling strategy for PET‐CT imaging does not affect the transfection efficiency of mRNA and protein expression, which is expected to facilitate quantitative, precise and longitudinal tracking of mRNA drugs in large mammals. Similarly, Kirschman et al. bound NeutrAvidin with fluorophore‐labeled oligos, which were linked with the 3′ UTR of mRNA.^[^ ^197^ ^]^ After electroporation, this constructed multiply labeled tetravalent RNA imaging probes (MTRIPs) successfully visualized endogenous mRNA without interring the protein expression.

**FIGURE 15:**
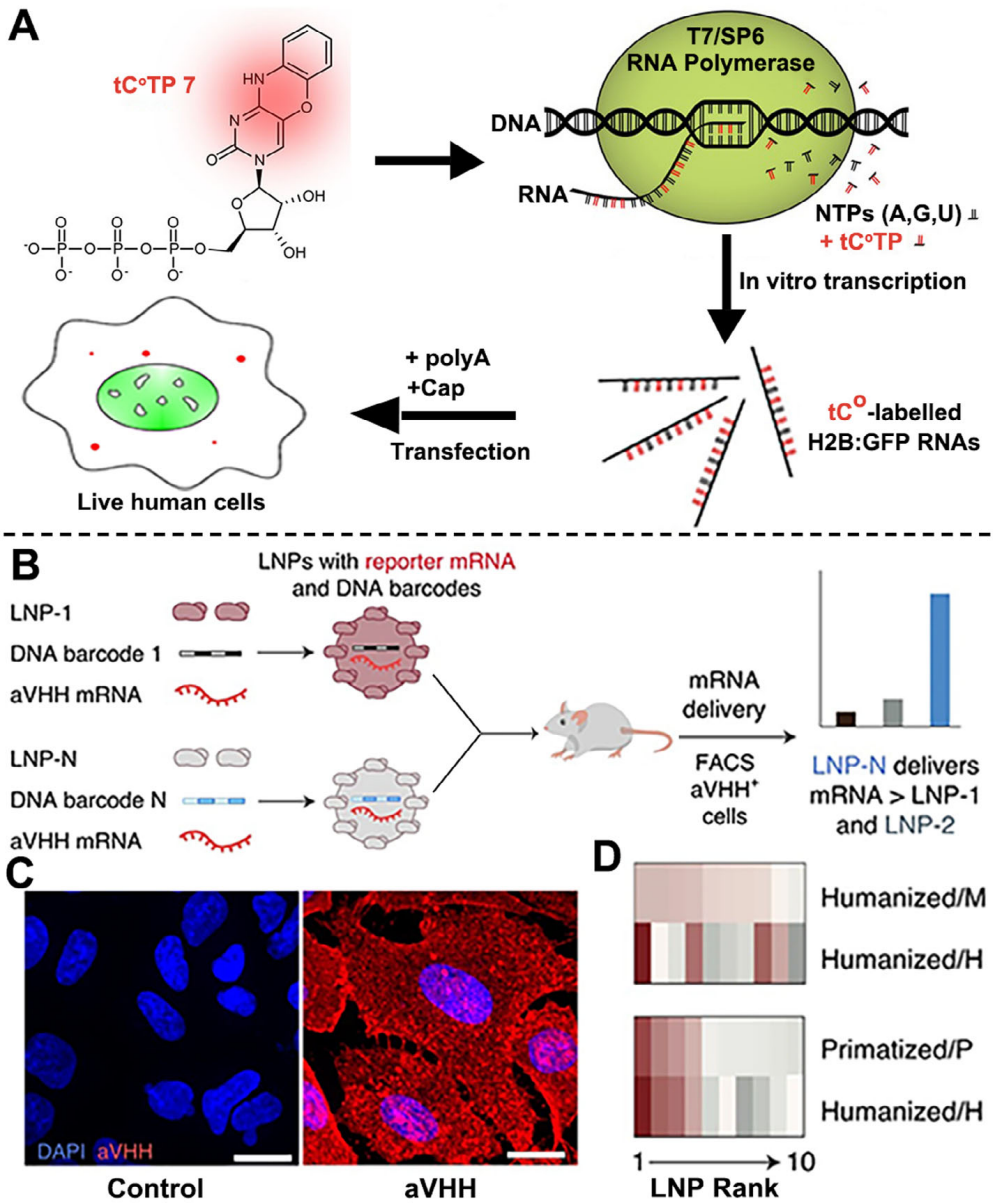
Live microscopy imaging of mRNA delivery in vivo and FACS‐mediated mRNA‐LNP screening. A Schematic illustration of enzymatic incorporating tC° in cytosine positions of natural mRNA and the translation of fluorescence‐labeled proteins. Reproduced with permission.^[^ ^198^ ^]^ Copyright 2021, Nature Publishing Group. B DNA barcode‐mediated identification of LNP and FACS‐mediated aVHH^+^ cells isolation. C Immunofluorescent imaging of aVHH protein expression in A549 cells after mRNA‐loaded LNP transfection. D Assessment of LNP‐mRNA delivery in hepatocytes of different species. Reproduced with permission.^[^ ^201^ ^]^ Copyright 2022, Nature Publishing Group.

Furthermore, Baladi et al. reported a stealth labeling method for mRNA, which utilizes an enzymatic reaction to incorporate triphosphates of tC° (a fluorescent tricyclic cytosine analog) at the native cytosine position of the target mRNA.^[^ ^198^ ^]^ Spatiotemporal tracking of mRNA delivery in vivo was achieved without the need for adding any foreign luminescent molecules (Figure 15A). The mRNAs of histone H2B and GFP fluorescent proteins were combined to image the translation products of mRNAs. Compared with other strategies for labeling mRNAs with fluorescence dyes (e.g., Cy5), the method that labels mRNA with fluorescent base analogs of nucleic acid not only realizes the direct visualization of mRNA and translation products in living cells but also guarantees that the translation and folding of proteins are not disturbed by the labeling molecules.^[^ ^199^ ^]^ Besides, the report by Baladi et al. also showcased an economical approach for synthesizing tC°, which guarantees its popularization. Near‐infrared imaging via fluorescent dyes can also be applied to mRNA in vivo tracking. Xiong et al. combined NIR imaging of tumors with mRNA delivery by adding PEGylated BODIPY dyes to dendrimer‐based LNPs, which achieved efficient cancer diagnosis and treatment, holding the prospect of clinical transformation.^[^ ^200^ ^]^

Unlike direct visualization of mRNA delivery in vivo to screen mRNA‐based platforms, Hatit et al. creatively designed a high‐throughput screen method for identifying LNPs with cell tropism for mRNA delivery.^[^ ^201^ ^]^ Specifically, this fast identification of nanoparticle delivery (FIND) approach quantifies the delivery of mRNA by adding unique DNA barcodes into the mRNA‐loaded LNP. They delivered mRNA encoding a glycosylphosphatidylinositol (GPI)‐anchored camelid VHH antibody (aVHH) (Figure 15B), which can be detected with an anti‐aVHH antibody for quantifying the protein expressing level, thus investigating the delivery efficiency of LNP. Figure 15C shows that aVHH was successfully expressed and localized on the cell surface. Using the FIND method, they effectively measured the mRNA‐delivering efficiency in multiple cell types of over 89 LNPs and picked out 10 top LNPs with different mRNA‐delivery efficiency in different species‐derived hepatocytes (Figure 15D). The method will help researchers to identify LNPs with specific cell tropisms and accelerate the clinical translation of mRNA‐based LNP platforms.^[^ ^202^ ^]^

Evaluating the targeting ability of mRNA‐LNP is of great significance. Other property parameters like mRNA loading capacity in LNP also count greatly. As such, Li et al. developed a high throughput technique (named multi‐laser cylindrical illumination confocal spectroscopy) to assess the payload capacity of mRNA in LNP with a small sampling volume, which can be applied to the quality control in scaling up manufacturing of mRNA‐LNPs.^[^ ^203^ ^]^ Moreover, to obtain more pharmaceutical information on mRNA‐based platforms before animal trials, three‐dimensional coculture models of tumors may represent a superior approach to facilitating the study of mRNA therapy.^[^ ^204^ ^]^

## 6 CLINICAL TRANSLATION OF MRNA‐BASED CANCER IMMUNOTHERAPY

Since the FDA approved two mRNA preparations for COVID‐19 vaccination, numerous preclinical platforms of mRNA‐based cancer immunotherapy have sprung up in the past three years. It is noteworthy that dozens of mRNA‐based formulations against cancer are being tested in phase I/II clinical trials, which shows the potential of mRNA in cancer treatment and provides valuable clinical data for guiding preclinical mRNA‐related projects.^[^ ^205^ ^]^

For instance, BI1361849 (CV9202), an mRNA‐based cancer immunotherapy composed of protamine in combination with local radiation, has been evaluated in a phase Ib trial (NCT01915524), where antigen‐specific immune responses were detected.^[^ ^22^ ^]^ Another cancer immunotherapeutic system BNT112 consists of mRNA encoding five prostate cancer‐specific antigens that are respectively formulated with liposomes to construct serum‐stable RNA lipoplexes (RNA‐LPX),^[^ ^206^ ^]^ is now in a phase II study of prostate cancer (NCT04382898) in combination with ICB therapeutic cemiplimab. mRNA‐5671 cancer immunotherapeutic system is an mRNA‐based LNP vaccine targeting four of the most frequent KRAS mutations (G12D, G13D, G12C, and G12V). Patients with advanced or metastatic non‐small cell lung, colorectal or pancreatic cancer and KRAS variations are under a Phase I study using mRNA‐5671 alone or in combination with pembrolizumab (NCT03948763).^[^ ^207^ ^]^ As a professional APC, DCs constantly engulf surrounding cellular materials. Transfecting DCs with mRNA to treat cancer was the first mRNA‐based cancer immunotherapy to reach clinical testing.^[^ ^208^ ^]^ In a phase II clinical study (NCT00510133), patients with acute myeloid leukemia (AML) in remission were treated with GRNVAC1 (VAC1) mRNA DCs and generally showed appreciable tolerance.^[^ ^209^ ^]^ A list of clinical trials using mRNA‐based immunotherapy for cancers is summarized in Table 1. As shown in these examples, peptide‐based formulations and LNPs account for the most applied delivery systems for mRNA for cancer immunotherapy. However, all the trials are still in phase I/II, which necessitates acceleration of the clinical research of mRNA‐based cancer treatment, focusing on LNPs and peptide‐based platforms. For more detailed clinical applications of mRNA, please refer to this review article.^[^ ^205^ ^]^

**TABLE 1:** Summary of clinical studies of mRNA‐based cancer immunotherapy.

Name of product	CTrials.gov identifier	Payload/delivery platform	Cancer type	Study phase	Patients, *n*	Latest time	Ref.
ECI‐006	NCT03394937	Intranodal injection of nacked TriMix and TAAs‐mRNA	Resected melanoma	I	21	2021	[210]
IVAC MUTANOME	NCT02035956	Intranodal injection of nacked mRNA encoding individual mutant neoantigens	Advanced melanoma	I	15	2020	[211]
CV9202	NCT01915524	Protamine with local irradiation	NSCLC	I	26	2016	[212]
mRNA‐4157	NCT03897881	Mutated neoepitope mRNA‐LNP with pembrolizumab	Melanoma	II	157	2023	[213]
NCI‐4650	NCT03480152	Neoantigen‐specific mRNA‐LNP	Several tumors	I‐II	5	2020	[214]
mRNA‐5671	NCT03948763	mRNA‐LNP with pembrolizumab	Several tumors	I	70	2022	[215]
PNOC020	NCT04573140	Tumor mRNA and pp65 LAMP‐loaded LNP	Glioblastoma	I	28	2023	[216]
BNT111	NCT04526899	mRNA‐lipoplex with cemiplimab	Unresectable melanoma	I	180	2023	[217]
BNT112	NCT04382898	mRNA‐lipoplex cancer vaccine with cemiplimab	Prostate cancer	I‐II	115	2023	[218]
BNT113	NCT04534205	mRNA‐lipoplex with pembrolizumab	Head and neck cancer	II	285	2023	[219]
BNT116	NCT05142189	mRNA‐lipoplex with cemiplimab and docetaxel	NSCLC	I	80	2023	[220]
BI 1361849	NCT03164772	TAAs‐mRNA loaded protamine	Metastatic NSCLC	I‐II	61	2022	[206]
CV9104	NCT01817738	Antigen‐mRNA loaded protamine	Prostate cancer	I‐II	197	2017	[221]
CV9201	NCT00923312	Antigen‐mRNA loaded protamine	NSCLC	I‐II	46	2018	[222]
TERT‐mRNA	NCT01456065	Survivin‐peptide	Ovarian epithelial cancer	I	15	2013	[223]
GRNVAC1	NCT00510133	Autologous dendritic cell vaccine	AML	II	21	2019	[224]
mRNA‐transfected DC	NCT00929019	Dendritic cell vaccination	Uveal melanoma	I‐II	23	2018	[225]
mRNA‐4157	NCT03313778	mRNA‐LNP with pembrolizumab	Solid tumors	I	108	2023	[226]
RO7198457	NCT03289962	mRNA‐lipoplex with atezolizumab	Melanoma, pancreatic cancer, etc.	I	272	2023	[227]
V940 (mRNA‐4157)	NCT05933577	mRNA‐LNP with pembrolizumab	Melanoma	III	1089	2023	[228]

Abbreviations: AML: acute myelogenous leukemia; LAMP: lysosomal associated membrane protein; NSCLC: non‐small cell lung cancer; TAAs: tumor‐associated antigens.

## 7 SUMMARY AND PERSPECTIVE

mRNA represents a promising approach for producing desired proteins, the main undertaker of life activities, which means a perfect supplement for all kinds of deficient molecules in disease occurrence and development. Additionally, mRNA possesses several superiorities as drug molecules, such as the convenience of IVT‐mRNA manufacturing, the safety of mRNA regarding avoidance of genome integration, and the amplifying efficacy of small dosages of mRNA. On the other hand, cancer is derived from cells that run out of the control of the body's immune system, which is partly associated with the negative loss of gene expression and the declining level of immunity. Therefore, the positive supplement of desired proteins via mRNA is just enough to offset the negative deficiency of functional substances in cancer.

However, several bottlenecks are limiting the clinical application of mRNA‐based therapeutics. First, the prerequisite for protein translation of mRNA is delivering it into the cytoplasm of target cells, where ribosomes, enzymes, and amino acids co‐exist. Moreover, the desired proteins must be expressed, secreted, or distributed in specific cell types in certain organs to perform a sufficient function and minimize off‐target side effects. Hitherto, the instability of mRNA structure, the low transfection and endosomal escape efficiency of mRNA, and the physiological barrier of transporting mRNA to specific cells restrict the clinical translation of mRNA‐based cancer immunotherapy. It is therefore necessary to develop proper strategies to overcome these bottlenecks from the perspective of modifying mRNA structure itself and designing novel delivery vectors with additional functionalities like self‐adjuvant, targeting, and endosomal escape ability.

Recently, with the approval of two mRNA‐based vaccines against COVID‐19 infection, the in vivo delivery of mRNA has emerged as a booming direction for curing various diseases like cancer.^[^ ^229^ ^]^ Tremendous reports on pushing mRNA into the clinical prevention of cancer are emerging. Modification of IVT‐mRNA structure, nucleoside modification, and codon optimization have become universal methods for enhancing its stability and transfection efficiency. Nearly all the delivered mRNA that appeared in the reviewed reports are modified as above, which means other novel strategies for mRNA construction should be emphasized. From our perspective, the concept of hybrid RNA may represent a breakthrough point for the future mRNA industry. For instance, co‐delivery of the hybrid product of mRNA and siRNA is expected to achieve the expression of deficient proteins and simultaneously silence the over‐expressed genes in tumor cells, which combines two anti‐tumor mechanisms and may exhibit appreciable performance in the clinic. Another strategy for mRNA‐based cancer immunotherapy mentioned in this review is harnessing adjuvants to boost immune response. We summarized several adjuvants applied in mRNA‐based treatment like the classical agonists of TLRs and STING genes, and there is still a plethora of reported adjuvants, which were not mentioned. Many organic or inorganic substances that possess immune system‐stimulating properties are being studied.^[^ ^61a^ ^]^ We highlighted the necessity of adding adjuvant in mRNA‐based cancer immunotherapy, which is crucial for the maturation and antigen‐presentation of APCs. The future adjuvant study in mRNA therapeutics is committed to digging out molecules with more potent immune‐stimulating properties and clinical safety.

Concerning the most prevalent strategy of mRNA therapeutics and delivery system design, we emphatically introduced the four well‐studied mRNA carriers such as LNP, gel‐like materials, polymer‐ and peptide‐based platforms. These delivery systems were designed to accommodate mRNA's unstable, negatively charged, and macromolecular properties and for cell‐targeted delivery. In terms of the supplementary idea of co‐delivering functional molecules like ICB therapeutic agents and pH‐responsive regions, it is the authors’ innovation point for enhancing mRNA‐based antitumor efficacy. The most important aspect of building an mRNA‐delivering system is endowing it with organ‐ and cell‐targeted properties, which ensure the desired proteins are expressed in specific cell types to play a function. The cell‐targeted delivery of mRNA represents an explanation of precision medicine, a clinical bottleneck of mRNA application in cancer treatment.

We summarized the attempts for precise transportation of mRNA to specific organs and cells such as tumor cells, DCs, T cells, B cells, and macrophages. First, a library of synthesized lipids can be constructed and the compositions of NPs can be optimized by adding SORT molecules to control their cell tropism. During this process, the typical structure of lipids that possess different cell tropisms can be discovered, which in combination with the elucidation of structure‐functionality mechanisms will present a robust approach for the systematic delivery of mRNA. Second, modifying the polymers with additional functional units to adjust its property like hydrophobicity and charge, can also alter the in vivo behavior of delivered mRNA. Third, the delivery platform can be modified with targeting moieties that can bind specifically to receptors that are highly expressed on target cells (e.g., Mannose‐modified NPs can target CD206 on M2 macrophages), which possesses attractive targeting efficiency but higher cost. Moreover, proper systemic and local administration routes play a crucial role in the targeted delivery of mRNA. Among these targeting strategies, we thought highly of exploring new lipids and lipid ratios in mRNA‐LNP platform and applying local administration routes for targeted delivery of mRNA, which is more conducive to clinical transformation. Future studies may concentrate on discovering the matching relationship between cell receptors and agents that can be easily attached to the delivery system. Apart from the strategies discussed above, Jiang et al. creatively designed a programmable RNA‐sensing technology, reprogrammable ADAR sensors, to achieve the cell‐specific delivery of mRNA.^[^ ^207^ ^]^ This method gates the translation of a cargo mRNA by adenosine deaminases acting on RNA (ADAR), which deactivates the stop codon upstream of aimed mRNA in the presence of specific endogenous RNA transcripts in target cells. This synthetic biological system pioneered a new approach for mRNA‐targeted delivery and will expedite the clinical translation of mRNA‐based cancer immunotherapy.

Among all these strategies described above, we highlighted the tissue‐ or cell‐targeted delivery of hybrid mRNAs with lipid‐ or polymer‐based vehicles, which are more economically manufacturable and enable better control over toxic side effects. With lower factory cost of mRNA and synergetic functions of proteins expressed by hybrid mRNAs, this platform possesses greater potential to enter clinical trials in the next five years for cancer immunotherapy. In addition to mRNA, nucleic acid drugs like circular RNA^[^ ^230^ ^]^ and self‐amplifying RNA^[^ ^231^ ^]^ are receiving increasing attention from researchers because of their stable structure, self‐replicating and self‐adjuvant properties. The breakthrough of nucleic acid drugs may be in these novel RNAs.

Notably, for clinical translation of mRNA‐based therapeutics, the long‐term storage of these formulations should also be emphasized. From another perspective, optimal storage conditions sometimes play a more crucial role in the popularization of commercial mRNA products than the original design process. Taking the most general LNP as an example, Zhao et al. investigated a series of conditions, such as temperature and physical states, for the long‐term storage of LNP‐mRNA.^[^ ^232^ ^]^ They found that in the liquid nitrogen storage condition, adding 5% (w/v) sucrose or trehalose to lipid‐like NPs is critical for the maintenance of mRNA delivery efficiency. Besides, lyophilization of mRNA‐LNP platforms can retain the stability and high antigenicity of mRNA at 25 °C over six months, thus dramatically improving the accessibility of mRNA‐based therapeutics in remote areas.^[^ ^233^ ^]^ Apart from LNP, Badieyan et al. used collagen matrix to deliver mRNA and this platform remained stable for at least six months at room temperature.^[^ ^142^ ^]^ As for other mRNA‐based formulations, investigating the optimal storage conditions is essential for clinical translation. Another point that requires consideration for the clinical application of mRNA therapeutics is that patients with different cancer subtypes may respond dissimilarly to the same mRNA system,^[^ ^10b^ ^]^ which impels researchers to customize personalized mRNA therapy for specific patient populations. Some recent clinical advances in Table 1 showed great potential for personalized mRNA neoantigen vaccines in treating solid tumors, pancreatic cancer, etc.^[^ ^27^, ^234^ ^]^ In summary, studies on intracorporal targeted mRNA delivery and protein expression to elicit robust immune response have demonstrated the prosperous future of mRNA therapeutics. The clinical translation of mRNA‐based cancer immunotherapy will benefit from deep integration of biochemistry in mRNA design, while employing materials and nanotechnology in targeted mRNA delivery.

## CONFLICT OF INTEREST STATEMENT

The authors declare no conflicts of interest. Wei Tao is a member of the *Exploration* editorial board.
